# RNAi-directed knockdown induces nascent transcript degradation and premature transcription termination in the nucleus

**DOI:** 10.1038/s41421-021-00297-8

**Published:** 2021-09-07

**Authors:** Jin You, Zhenxing Song, Jiamei Lin, Ruirui Jia, Fei Xia, Zhengguo Li, Chuan Huang

**Affiliations:** 1 School of Life Sciences, Chongqing University, Chongqing, 401331 China; 2 Center of Plant Functional Genomics, Institute of Advanced Interdisciplinary Studies, Chongqing University, Chongqing, 401331 China

**Keywords:** Biological techniques, RNAi

Dear Editor,

As one of the most popular technological breakthroughs, RNA interference (RNAi)-directed transcript knockdown is a rapid, efficient, specific, and potentially high-throughput approach that is not only broadly used in the fields of protein and RNA biology for various experimental purposes, but also provides a therapeutic strategy for widespread clinical/biomedical applications^1^. To achieve RNAi-directed knockdown, synthetic small interfering RNAs (siRNAs) or long double-strand RNAs (dsRNAs; siRNA precursors) can be designed to harness the Argonaute-mediated mechanism as well summarized by Kawamata and Tomari^2^. RNAi typically modulates gene expression post-transcriptionally in the cytoplasm; however, it is poorly understood whether exogenous dsRNAs/siRNAs could affect RNA polymerase II (Pol II) transcription of targeted genes in the nucleus. If so, it will be much more challenging to conclude a *cis*-acting function for the protein produced or the transcript itself that regulates expression of the encoded gene. Therefore, the raised question is extremely important for the appropriate application of RNAi, and more evidence is needed to fill the gap.

In this study, three protein-coding transcripts (*MtnA*, *dati*, and *Hsp70Aa*) and a well-characterized lncRNA *roX1* were selected to evaluate the effect of RNAi on targeted gene transcription based on their variable lengths, cytoplasmic or nuclear subcellular localization, and the presence or absence of introns (Supplementary Fig. S1a, b). To confirm the knockdown efficiency of each dsRNA, *Drosophila* Schneider 2 (S2) cells were transfected with the indicated dsRNAs for 2 days. Real-time RT-PCR was performed to examine expression of the steady-state transcript. As expected, these dsRNAs are able to efficiently reduce expression of the targeted genes post-transcriptionally (Supplementary Fig. S1c–f). Intriguingly, the pre-mRNA of the targeted gene was also silenced by RNAi (Supplementary Fig. S1g, h), providing a hint that RNAi strategy itself may influence transcriptional activity of the targeted gene. To further investigate whether RNAi triggers degradation of newly-synthesized transcripts, dsRNA-treated cells were labeled with 4-Thiouridine (4sU) followed by 4sU-labeled RNA purification (Supplementary Fig. S2a). Real-time RT-PCR was performed to examine the level of 4sU-labeled RNAs. Pre-mRNAs were robustly enriched in 4sU-labeled RNA samples (Supplementary Fig. S2b), demonstrating that the phenotype in metabolic labeling experiments was not caused by steady-state RNA contamination. We surprisingly found that dsRNA transfection led to a ∼50–80% decrease in expression of the 4sU-labeled transcripts regardless of whether they were analyzed with primer sets positioned upstream or downstream of the dsRNA targeting site (Fig. 1a, b; Supplementary Fig. S2c–e). Transcription of the reference gene *rp49* was largely unaffected (Fig. 1b; Supplementary Fig. S2c–e). Considering that metabolic incorporation of the labeled nucleotide in intact cells may have adverse effects on cell physiology, we performed nuclear run-on (NRO) assays with 5-Bromouridine 5’-triphosphate (BrUTP) to ascertain the phenotype (Supplementary Fig. S3a, b). Similarly, dsRNA transfection induced a robust reduction in the nascent RNA abundance of the targeted genes in NRO samples (Fig. 1a, c; Supplementary Fig. S3c–e). Next, we asked whether RNAi affects Pol II recruitment to the targeted locus by chromatin immunoprecipitation (ChIP) experiments (Supplementary Fig. S4a). Interestingly, RNAi resulted in dissociation of Pol II from the region downstream, but not upstream, of the dsRNA targeting site (Fig. 1d; Supplementary Fig. S4b–d). Pol II recruitment to the *rp49* locus was largely unaffected in each sample (Supplementary Fig. S4e). This suggests that exogenous dsRNA/siRNA stimulates premature termination by releasing Pol II from the region downstream of the dsRNA targeting site. Taken together, although transcripts have variable lengths, sequences, translation capacity, and localization, their transcriptional activities can be impaired by RNAi.

**Fig. 1:**
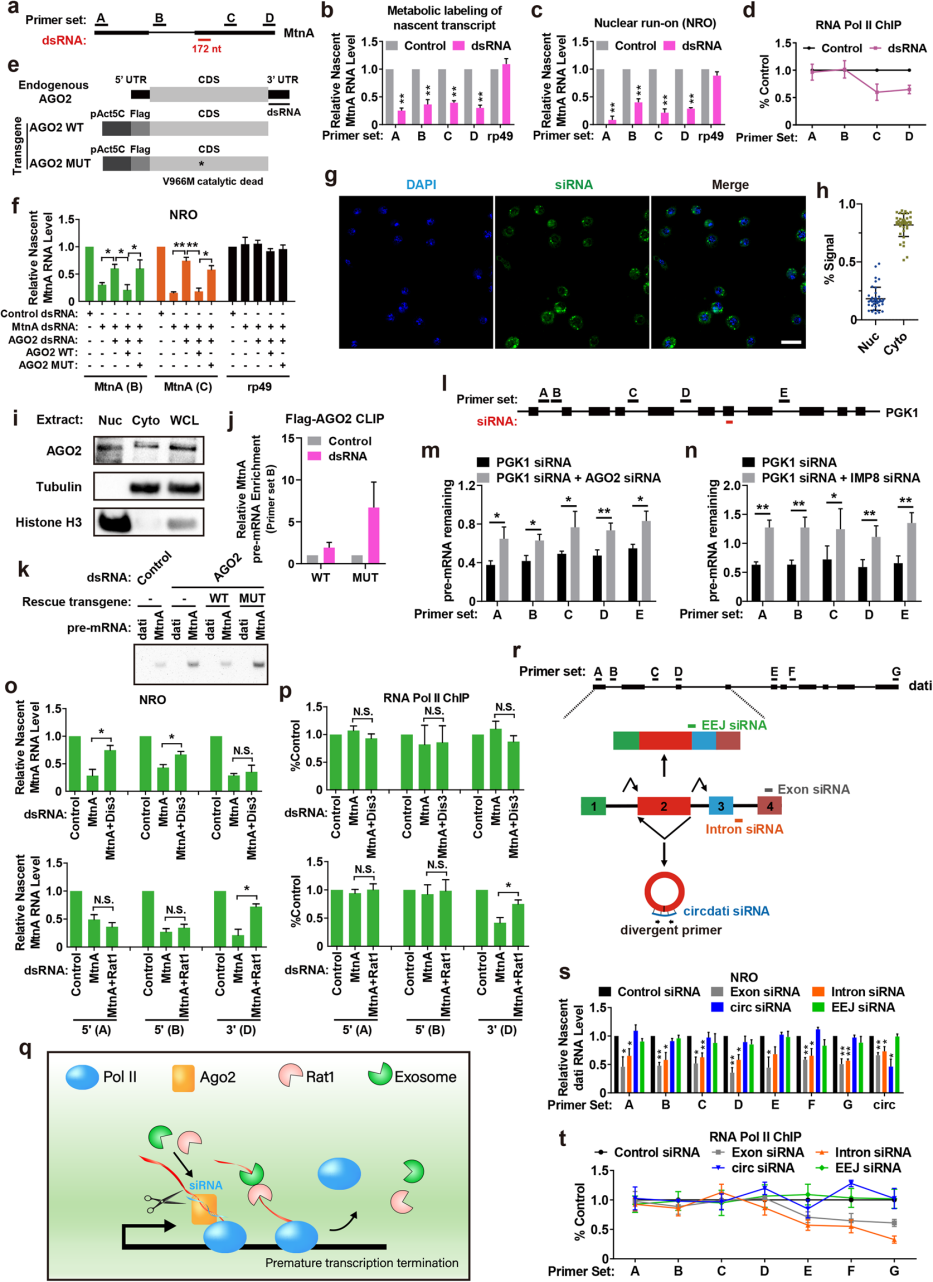
RNAi induces premature transcription termination. **a** The *Drosophila MtnA* locus with the locations of PCR amplicons and dsRNA targeting site. Real-time RT-PCR analysis of *MtnA* nascent transcript level in metabolic labeling (**b**) or NRO (**c**) experiments. **P < 0.01; *P < 0.05. **d** Real-time ChIP-PCR analysis of Pol II occupancy at the *MtnA* locus. **e** Schematic of Ago2 knockdown/rescue strategy. **f** Real-time RT-PCR analysis of *MtnA* nascent transcript level in Ago2 knockdown/rescue experiments. **P < 0.01; *P < 0.05. **g** Subcellular localization of FAM-labeled *dati* siRNAs. Representative images are shown. Scale bars, 15 μm. **h** Statistics of FAM-labeled siRNA signal in the nucleus and cytoplasm. *n* = 40 cells for calculation. **i** Western blotting analysis of Ago2 in nuclear, cytoplasmic, or whole cell extract. Tubulin and Histone H3 served as a cytoplasmic and nuclear marker, respectively. Representative blots are shown. **j** Real-time RT-PCR analysis of *MtnA* pre-mRNA enrichment in Flag-tagged Ago2 crosslinking IP (CLIP) samples. **k** RT-PCR analysis of *MtnA* pre-mRNA enrichment in *MtnA* siRNA pulldown samples. **l** The human *PGK1* locus with the locations of PCR amplicons and siRNA targeting site. Real-time RT-PCR analysis of *PGK1* pre-mRNA level in Ago2 (**m**) or IMP8 (**n**) knockdown cells. **P < 0.01; *P < 0.05. **o** Real-time RT-PCR analysis of *MtnA* nascent transcript level in Dis3 (top) or Rat1 (bottom) knockdown experiments. **P < 0.01; *P < 0.05. **p** Real-time ChIP-PCR analysis of Pol II occupancy at the *MtnA* locus in Dis3 (top) or Rat1 (bottom) knockdown experiments. **P < 0.01; *P < 0.05. **q** The working model of RNAi-induced premature termination. **r** The *dati* locus with the locations of PCR amplicons (top). The model of pre-mRNA splicing and the locations of siRNA targeting sites (bottom). **s** Real-time RT-PCR analysis of *dati* nascent transcript level following siRNA transfection. **P < 0.01; *P < 0.05. **t** Real-time ChIP-PCR analysis of Pol II occupancy at the *dati* locus following siRNA transfection. Data are shown as means ± SEM of three independent replicates and were normalized to the control dsRNA (**b-d**, **f**, **j**, **o**, **p**) or siRNA (**m**, **n**, **s**, **t**) sample.

Given that RNA cleavage in pre-mRNAs has been shown to exert an important role in Pol II premature termination^3^ and *Drosophila* Ago2 is indispensable for siRNA-guided RNA cleavage^4^, we reasoned that Ago2 may function in exogenous dsRNA/siRNA-induced premature transcription termination. To this end, two *Ago2* transgenes (wild-type and catalytically dead) that only harbor the coding sequence of *Ago2* and an *Ago2* dsRNA targeting the 3’UTR were used for the rescue assay. *Ago2* 3’UTR dsRNA only depletes endogenous *Ago2* but not transgene-derived *Ago2* (Fig. 1e). S2 cells were transfected with the control or *Ago2* dsRNA for 1 day, and dsRNA targeting *MtnA*, *dati*, *Hsp70Aa*, or *roX1* was introduced for another 2 days. NRO experiments were performed followed by real-time RT-PCR. In Ago2-knockdown cells, RNAi no longer resulted in a robust reduction in the nascent transcript abundance of the targeted gene (Fig. 1f; Supplementary Fig. S5a–c). Reexpression of a wild-type Ago2 significantly attenuated transcription, whereas reexpression of a catalytically dead Ago2 had little effect (Fig. 1f; Supplementary Fig. S5a–c). The role of Ago2 in nascent RNA degradation was also confirmed using Ago2*-*knockout cells (Supplementary Fig. S5d, e). Moreover, exogenous siRNAs and Ago2 were found to be partially nuclear-localized in S2 cells (Fig. 1g–i) and partially co-localized in the nucleus (Supplementary Fig. S6a). Ago2 depletion had little effect on the subcellular localization of exogenous siRNAs (Supplementary Fig. S6b, c), but RNAi promoted Ago2 nuclear translocation (Supplementary Fig. S7) and triggered Ago2 recruitment to the targeted pre-mRNA (Fig. 1j). We noticed that the targeted pre-mRNA showed a higher enrichment in catalytically dead Ago2 IPs over wild-type Ago2 IPs. Similarly, the interaction between siRNA and its targeted pre-mRNA was enhanced in Ago2-knockdown cells or catalytically dead Ago2-rescued cells (Fig. 1k). It is likely that cleavage of the targeted RNA may trigger the release of Ago2. Next, we tested whether RNAi affects transcription in HeLa cell line (Fig. 1l–n; Supplementary Fig. S8). Likewise, *PGK1* siRNA transfection led to a 30%–50% decrease in the level of *PGK1* pre-mRNA, and depletion of Ago2 or Importin 8, an Ago2 nuclear import factor^5^, abolished the effect (Fig. 1m, n; Supplementary Fig. S8). Together, these results support a model in which Ago2 is rapidly recruited by exogenous siRNA to cleave the targeted nascent RNA, thereby promoting premature termination in the nucleus.

RNA quality control is a process that is tightly linked to transcription. RNP assembly defect or RNA cleavage triggers RNA degradation within the site of transcription by 5’ and 3’ exonuclease^3^. Considering that Ago2-mediated RNA cleavage generates a free 5’ end of the downstream fragment and a free 3’ end of the upstream fragment, we hypothesized that these fragments may provide an entry point for 5’ degradation by Rat1 or 3’ degradation by Dis3. To test our hypothesis, S2 cells were transfected with the control, *Dis3*, or *Rat1* dsRNA for 1 day, and dsRNA targeting *MtnA*, *dati*, *Hsp70Aa* or *roX1* was introduced for another 2 days. NRO experiments were then performed followed by real-time RT-PCR. As expected, the level of the upstream fragment was rescued upon Dis3 depletion, and the level of the downstream fragment was restored to a level similar to the control sample in Rat1-depleted cells (Fig. 1o; Supplementary Fig. S9). Furthermore, Pol II recruitment to the targeted locus was also examined by ChIP experiments, showing that Rat1 is required for Pol II release while Dis3 has almost no effect on Pol II occupancy (Fig. 1p; Supplementary Fig. S9). This mirrors a “torpedo” model for transcription termination^3^, in which the upstream RNA has already been transcribed by Pol II, while Pol II of the downstream RNA is caught up and released by Rat1 (Fig. 1q).

The *cis* effect of a linear lncRNA on its encoded gene transcription is now becoming increasingly an important field of gene regulation; however, informed by Fig. 1a–q, RNAi strategy itself may cause premature transcription termination of the targeted locus. Therefore, the related conclusion based on this technique might be challenging and could be carefully reconsidered. It is important to note that thousands of protein-coding genes generate not only linear RNAs but also circular RNAs (circRNAs) via backsplicing^6^ (Fig. 1r). Emerging studies are raised to investigate the functions of circRNAs using RNAi strategy. Despite circRNA-targeted siRNA (circ-siRNA) being typically designed to target circular junction (Fig. 1r), it is still unknown whether circ-siRNA triggers premature transcription termination, since circ-siRNA is partially base-paired to the encoded pre-mRNA. To fill the gap, a circ-siRNA was designed to target the circular junction of *circdati*, a well-characterized circRNA as described in our previous study^7,8^. Transcriptional activity and Pol II occupancy of the *dati* locus were examined by NRO and Pol II-ChIP assay, respectively. In cells transfected with *circdati* siRNA, the level of nascent *circdati* was drastically decreased, whereas the level of nascent *dati* mRNA was largely unaffected (Fig. 1s), suggesting that circ-siRNA does not impair production of the encoded linear RNA. Additionally, *circdati* siRNA transfection had little effect on Pol II occupancy at the *dati* gene body (Fig. 1t). These data suggest that circ-siRNA does not impair transcriptional activity of the encoded gene; therefore, RNAi strategy may be used to establish an RNA-mediated function for a circRNA locus. Informed by this, we further examined whether a siRNA against exon-exon junction (EEJ) is capable of getting around the pitfall of siRNAs. As observed, EEJ siRNA significantly reduced the level of mature *dati* mRNA (Supplementary Fig. S10), but had almost no effect on *dati* transcription (Fig. 1s, t). Therefore, we concluded that RNAi-induced nascent transcript degradation may be avoided by targeting EEJ.

Although RNAi machinery was first thought to function solely in the cytoplasm, it also has crucial roles in some nuclear events, such as promoter methylation and heterochromatin formation^9^. On the other hand, RNAi-directed knockdown is a vitally important technique to suppress gene expression in eukaryotic system. Instead of investigating the genome-wide picture of RNAi-triggered disturbances, our study focuses on the unexpected effect of exogenous dsRNA/siRNA reagents on their targeted genes in *cis*. Mirroring the natural mechanism, RNAi attenuates generation of nascent transcripts by inducing premature termination which is combinatorially controlled by Ago2, Dis3, and Rat1 (Fig. 1q). In fact, similar phenotype has been observed a decade ago^10,11^. Guang et al. first demonstrated that endogenous siRNAs can be transported to the nucleus to pause RNA Pol II, inhibit transcription elongation, and cause premature termination in *Caenorhabditis elegans*^10,11^. Notably, they found that *lin-15b* pre-mRNA silencing accounted for ~80% of total *lin-15b* transcript silencing elicited by RNAi^10^. This suggests that siRNA may function predominantly in the nucleus to nascent transcripts in certain cases, which is consistent with our results (Fig. 1b, c). Unlike exon or intron siRNA, circ-siRNA and EEJ siRNA do not affect transcriptional activity of the encoded gene. It is probably due to the specificity of these siRNAs, merely targeting the junction site. Similar to RNAi, antisense oligonucleotide (ASO)-mediated transcript knockdown also triggers cleavage of nascent transcripts and efficient pre-mRNA degradation on chromatin^8,12,13^. Therefore, the unexpected effects of these strategies on gene expression should be taken into consideration before any experimental or clinical applications.

## Supplementary information

Supplementary Information
