# Supplementary material for: RNAi-directed knockdown induces nascent transcript degradation and premature transcription termination in the nucleus
Source: Cell Discov. 2021 Sep 7;7:79. doi: 10.1038/s41421-021-00297-8 (PMC8421446; doi:10.1038/s41421-021-00297-8)
Supplement: Supplementary file 1 — Supplementary Information [file 41421_2021_297_MOESM1_ESM.pdf]

Supplementary Information

RNAi-directed knockdown induces nascent transcript degradation and premature transcription termination in the nucleus

Jin You, Zhenxing Song, Jiamei Lin, Ruirui Jia, Fei Xia, Zhengguo Li, and Chuan Huang

Supplementary Figure S1-S10
Supplementary Figure Legends
Supplementary Methods
Supplementary References
Supplementary Table S1-S4
Supplementary Plasmid Information

Figure S1

a

Gene Name	Intron Count	Genomic Length (bp)	Mature Transcript Length (nt)	Subcellular Localization	Protein-coding or Non-coding
Hsp70Aa	0	2383	2383	Cytoplasm	Protein-coding
dati	9	17771	3937	Cytoplasm	Protein-coding
MtnA	1	965	700	Cytoplasm	Protein-coding
roX1	0	3758	3758	Nucleus	Non-coding

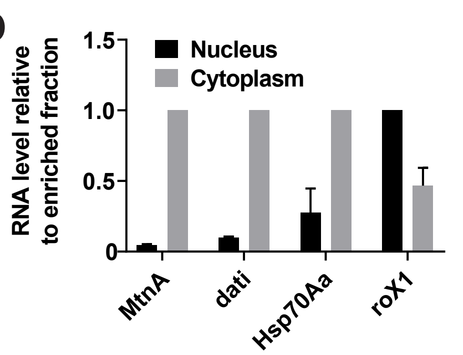

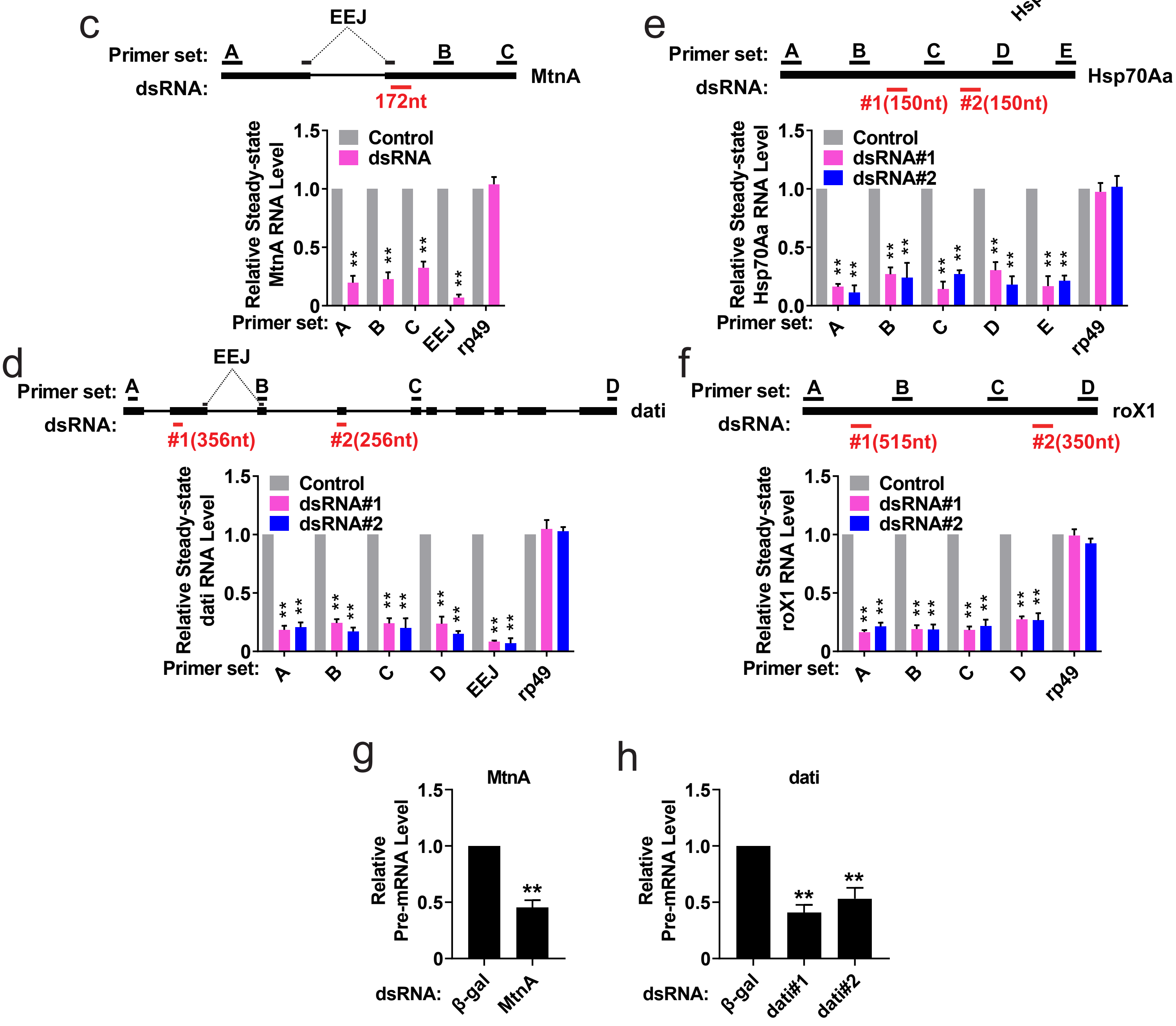

Figure S2

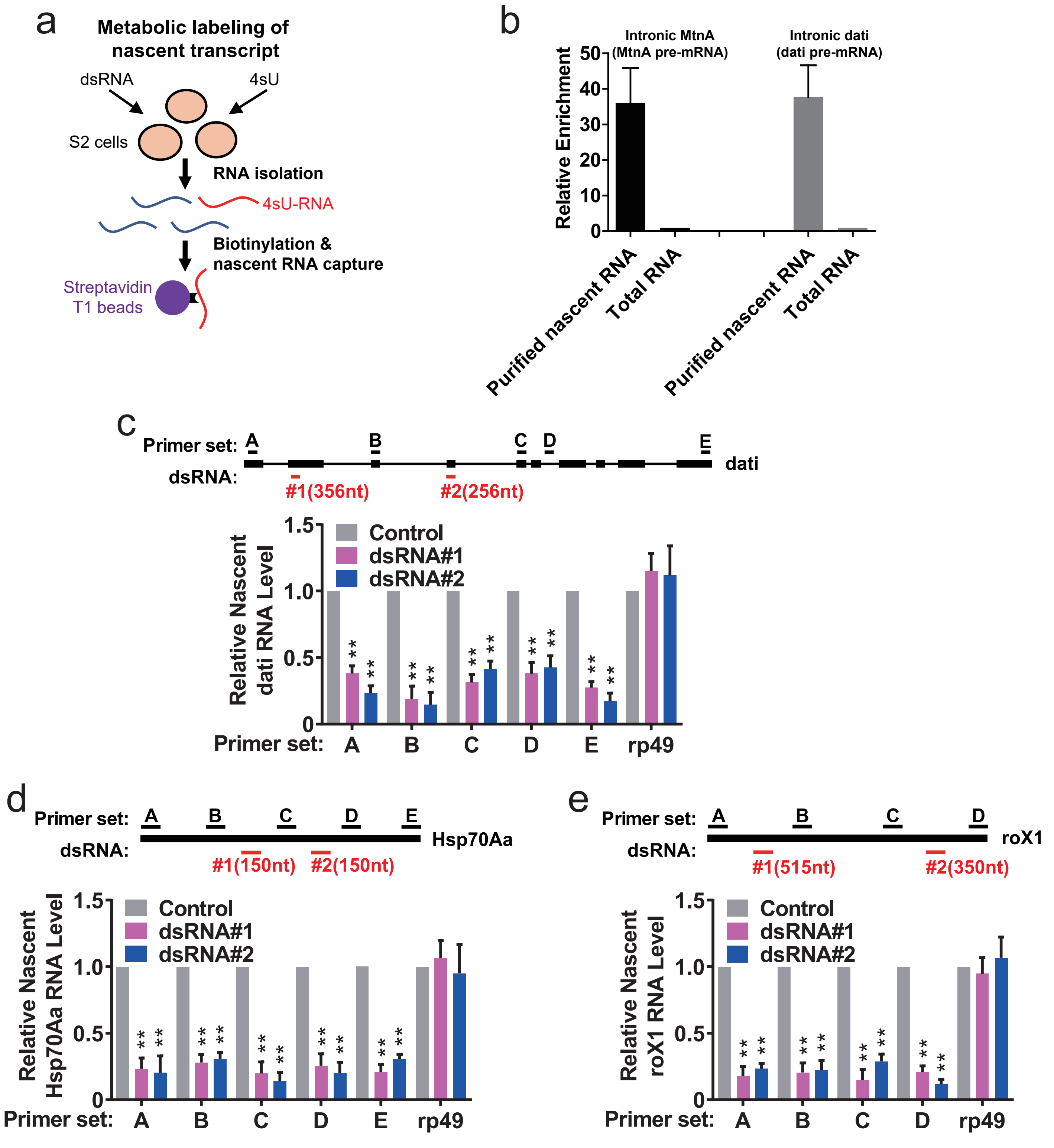
a
Metabolic labeling of nascent transcript
dsRNA
4sU
S2 cells
RNA isolation
4sU-RNA
Biotinylation & nascent RNA capture
Streptavidin T1 beads
b
Intronic MtnA (MtnA pre-mRNA)
Intronic dati (dati pre-mRNA)
Relative Enrichment
Purified nascent RNA
Total RNA
c
Primer set:
dsRNA:
dati
#1(356nt)
#2(256nt)
Control
dsRNA#1
dsRNA#2
Relative Nascent dati RNA Level
Primer set:
rp49
d
Hsp70Aa
#1(150nt)
#2(150nt)
Relative Nascent Hsp70Aa RNA Level
e
roX1
#1(515nt)
#2(350nt)
Relative Nascent roX1 RNA Level

Figure S3

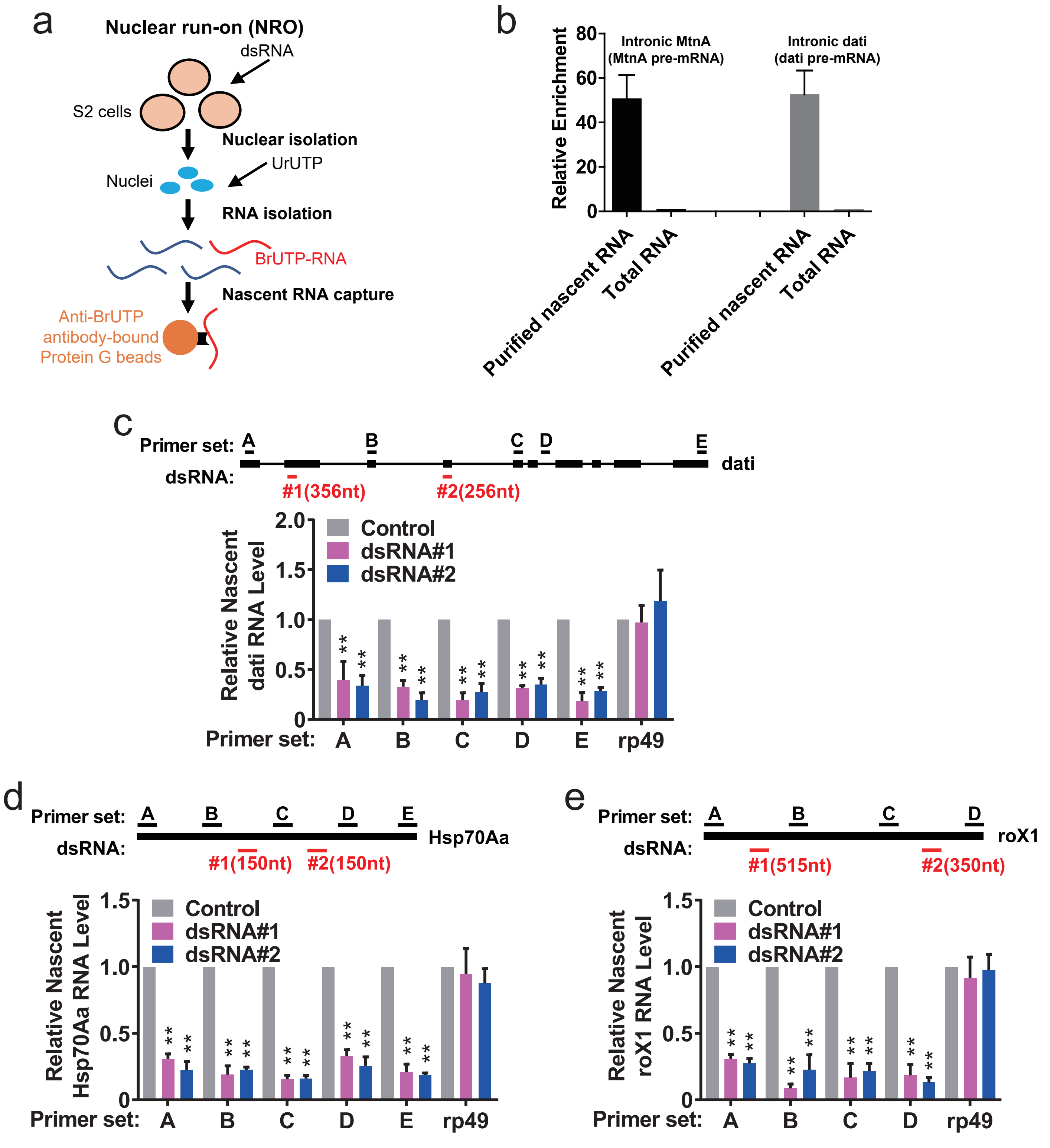
a
Nuclear run-on (NRO)
dsRNA
S2 cells
Nuclear isolation
UrUTP
Nuclei
RNA isolation
BrUTP-RNA
Nascent RNA capture
Anti-BrUTP antibody-bound Protein G beads
b
Relative Enrichment
Intronic MtnA (MtnA pre-mRNA)
Intronic dati (dati pre-mRNA)
Purified nascent RNA
Total RNA
c
Primer set: A B C D E
dsRNA: #1(356nt) #2(256nt)
dati
Relative Nascent dati RNA Level
Control
dsRNA#1
dsRNA#2
Primer set: A B C D E rp49
d
Primer set: A B C D E
dsRNA: #1(150nt) #2(150nt)
Hsp70Aa
Relative Nascent Hsp70Aa RNA Level
Control
dsRNA#1
dsRNA#2
Primer set: A B C D E rp49
e
Primer set: A B C D
dsRNA: #1(515nt) #2(350nt)
roX1
Relative Nascent roX1 RNA Level
Control
dsRNA#1
dsRNA#2
Primer set: A B C D rp49

Figure S4

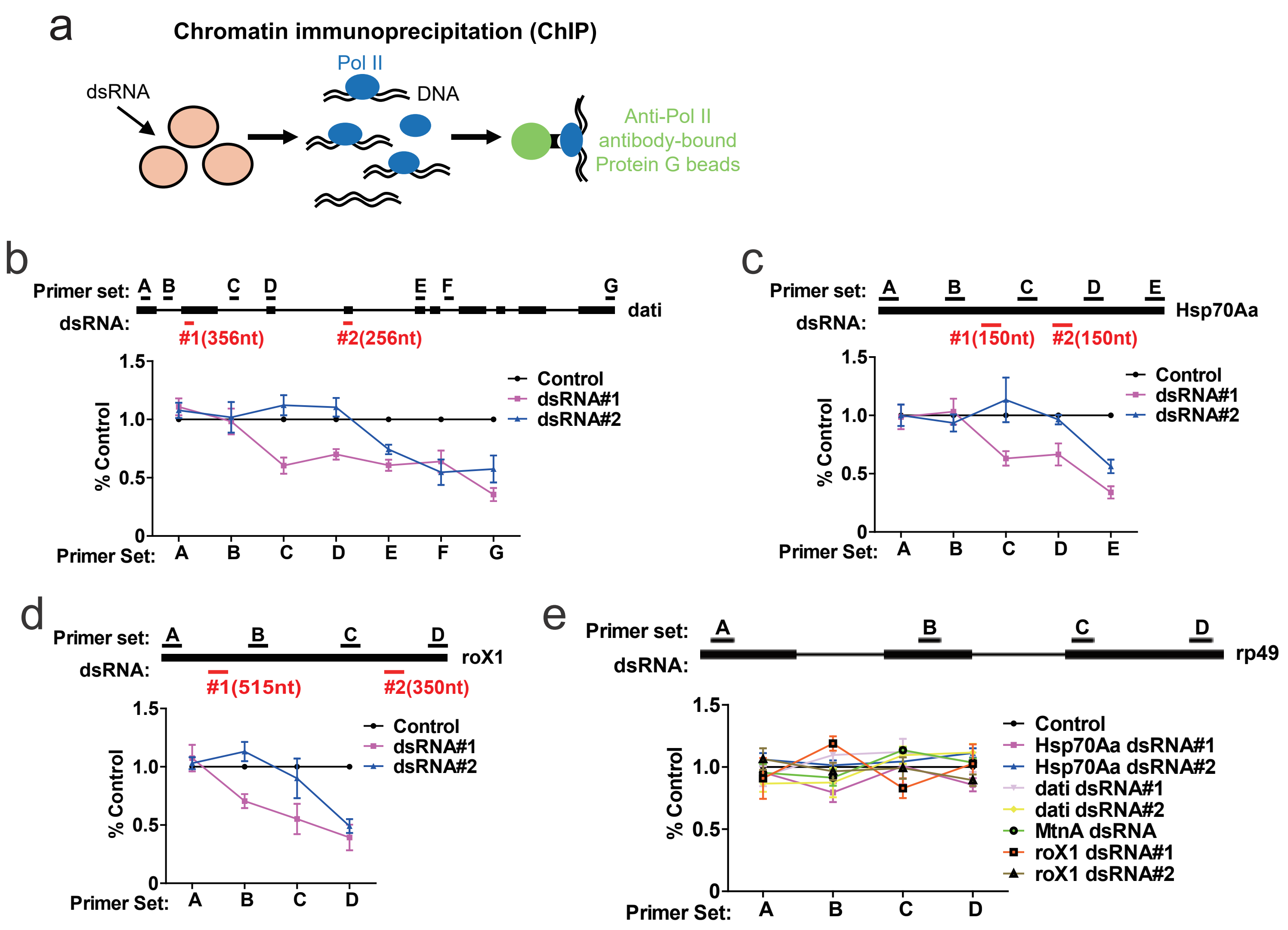
a
Chromatin immunoprecipitation (ChIP)
dsRNA
Pol II
DNA
Anti-Pol II
antibody-bound
Protein G beads
b
Primer set: A B C D E F G
dsRNA: #1(356nt) #2(256nt)
dati
% Control
Control
dsRNA#1
dsRNA#2
Primer Set: A B C D E F G
c
Primer set: A B C D E
dsRNA: #1(150nt) #2(150nt)
Hsp70Aa
% Control
Control
dsRNA#1
dsRNA#2
Primer Set: A B C D E
d
Primer set: A B C D
dsRNA: #1(515nt) #2(350nt)
roX1
% Control
Control
dsRNA#1
dsRNA#2
Primer Set: A B C D
e
Primer set: A B C D
dsRNA:
rp49
% Control
Control
Hsp70Aa dsRNA#1
Hsp70Aa dsRNA#2
dati dsRNA#1
dati dsRNA#2
MtnA dsRNA
roX1 dsRNA#1
roX1 dsRNA#2
Primer Set: A B C D

Figure S5

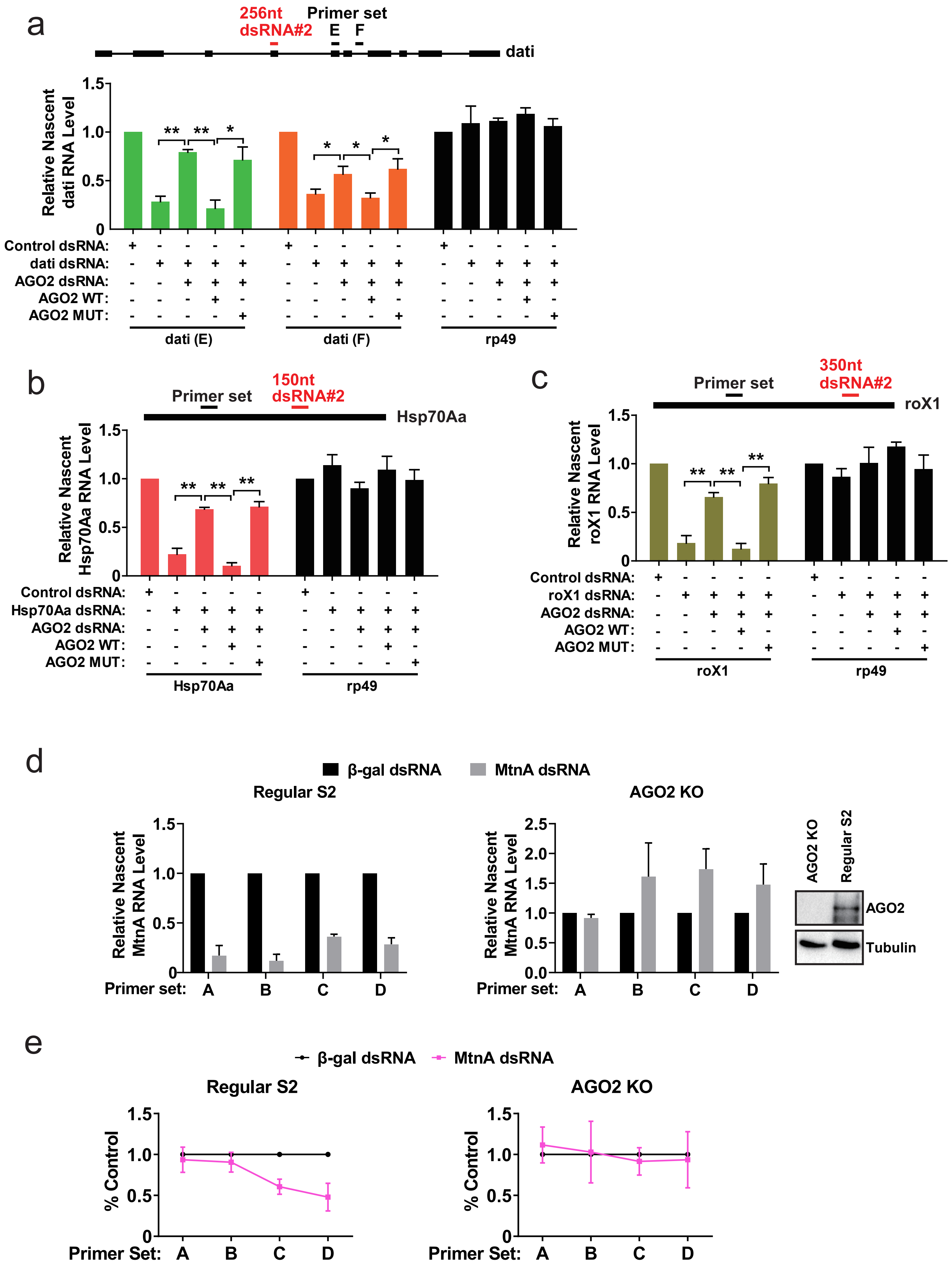

a
256nt dsRNA#2
Primer set
E F
dati
Relative Nascent dati RNA Level
1.5
1.0
0.5
0
** ** *
* * *
Control dsRNA:
dati dsRNA:
AGO2 dsRNA:
AGO2 WT:
AGO2 MUT:
dati (E)
dati (F)
rp49
b
Primer set
150nt dsRNA#2
Hsp70Aa
Relative Nascent Hsp70Aa RNA Level
Control dsRNA:
Hsp70Aa dsRNA:
AGO2 dsRNA:
AGO2 WT:
AGO2 MUT:
Hsp70Aa
rp49
c
Primer set
350nt dsRNA#2
roX1
Relative Nascent roX1 RNA Level
Control dsRNA:
roX1 dsRNA:
AGO2 dsRNA:
AGO2 WT:
AGO2 MUT:
roX1
rp49
d
β-gal dsRNA
MtnA dsRNA
Regular S2
AGO2 KO
Relative Nascent MtnA RNA Level
2.5
2.0
1.5
1.0
0.5
0.0
Primer set: A B C D
AGO2 KO
Regular S2
AGO2
Tubulin
e
β-gal dsRNA
MtnA dsRNA
Regular S2
AGO2 KO
% Control
Primer Set: A B C D

Figure S6

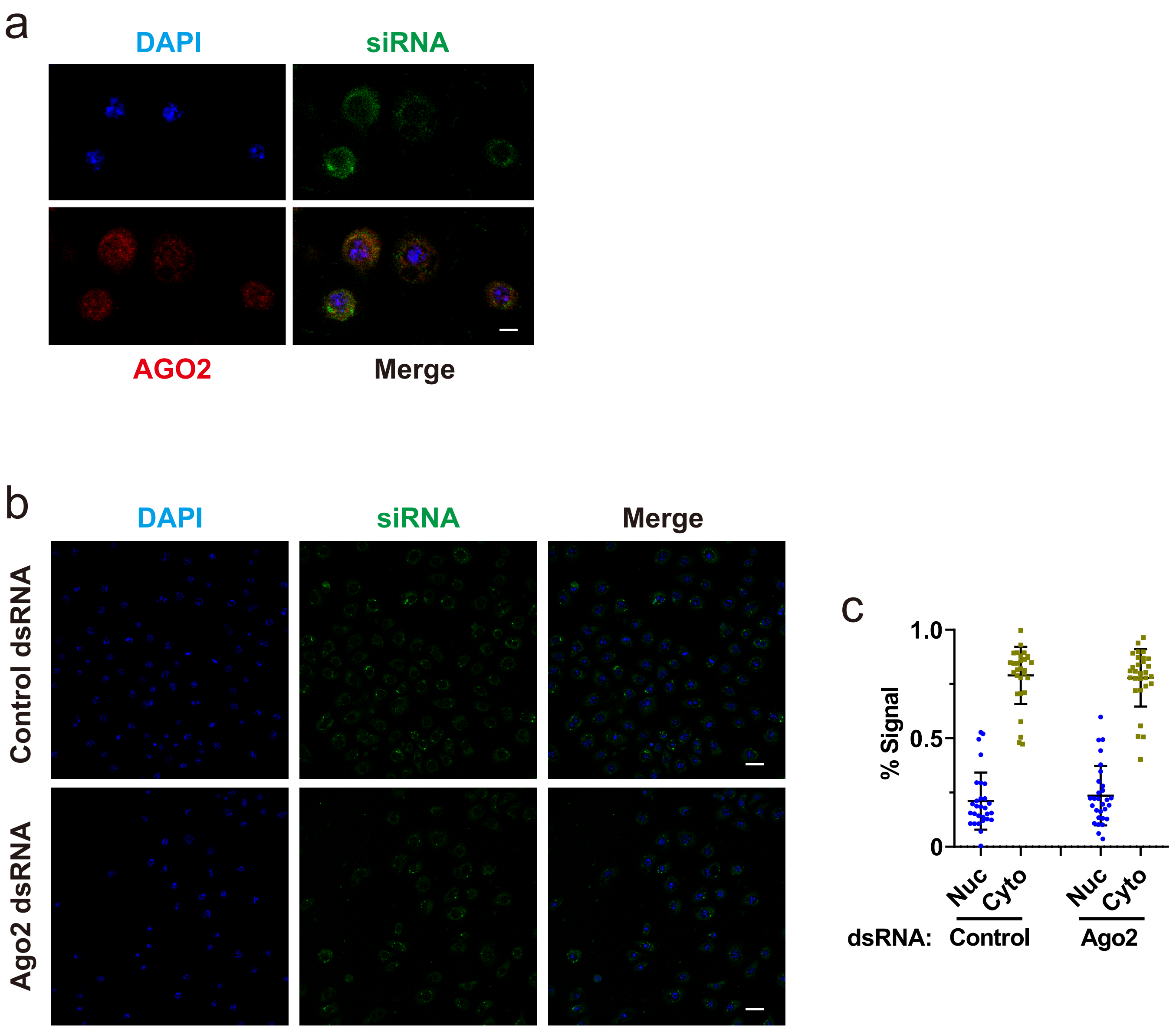
a
DAPI
siRNA
AGO2
Merge
b
DAPI
siRNA
Merge
Control dsRNA
Ago2 dsRNA
C
1.0
0.5
0
% Signal
Nuc
Cyto
Nuc
Cyto
dsRNA:
Control
Ago2

Figure S7

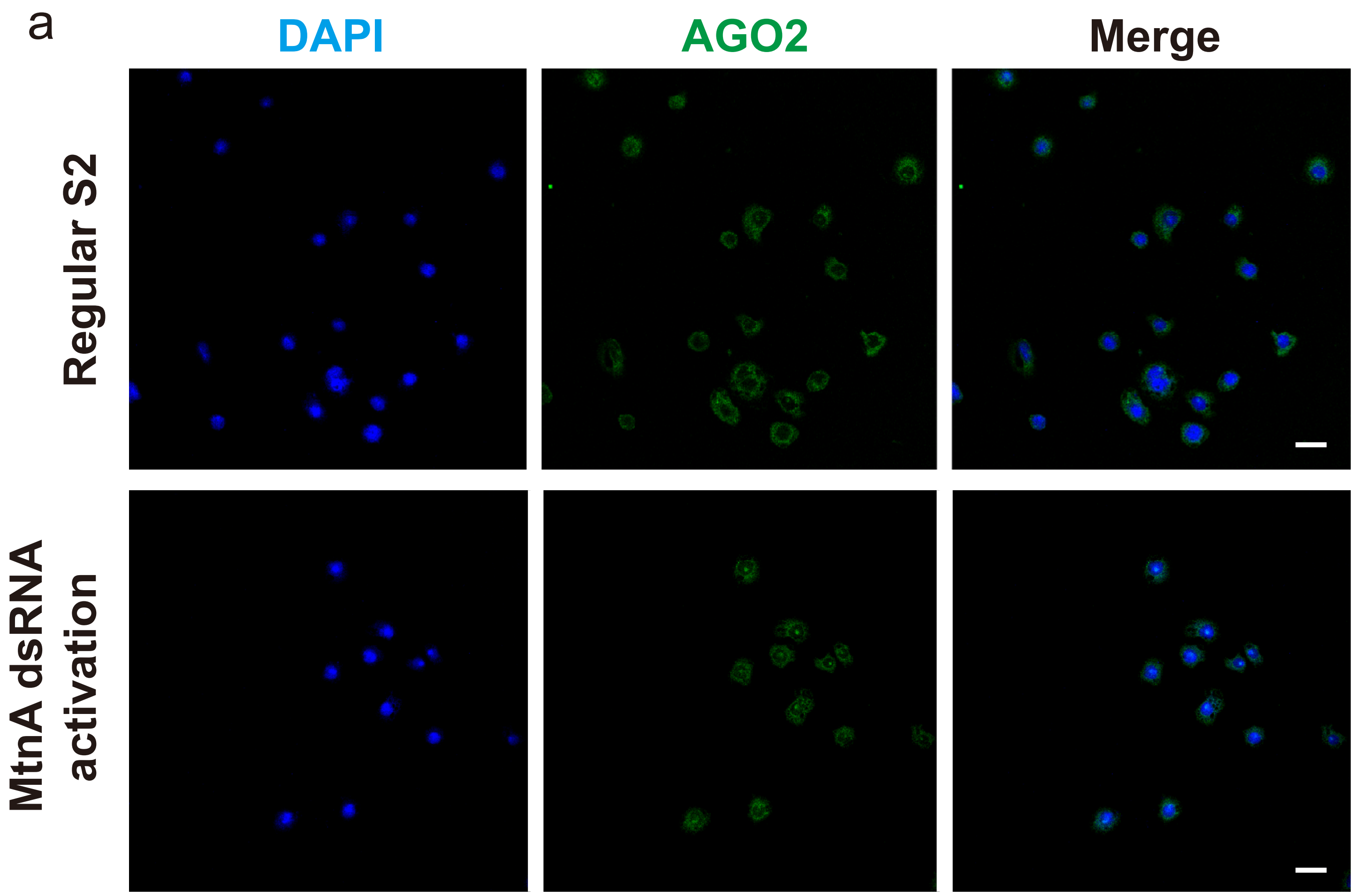

a
DAPI
AGO2
Merge
Regular S2
MtnA dsRNA activation

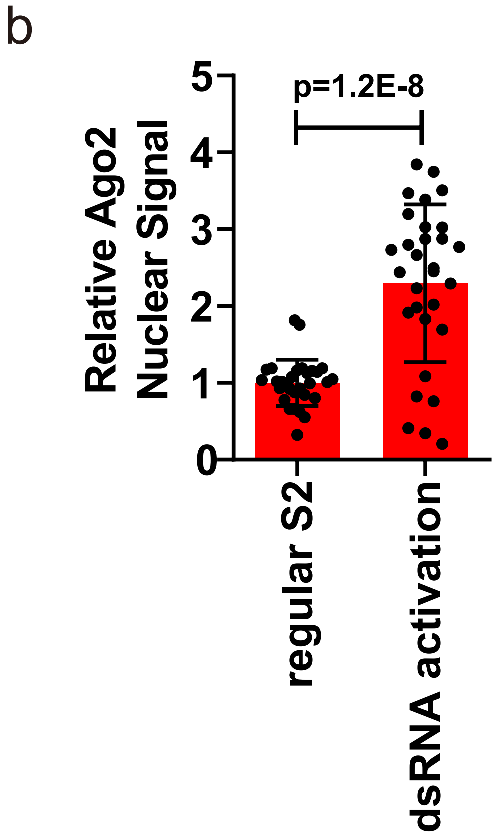

b
Relative Ago2 Nuclear Signal
p=1.2E-8
0
1
2
3
4
5
regular S2
dsRNA activation

Figure S8

a

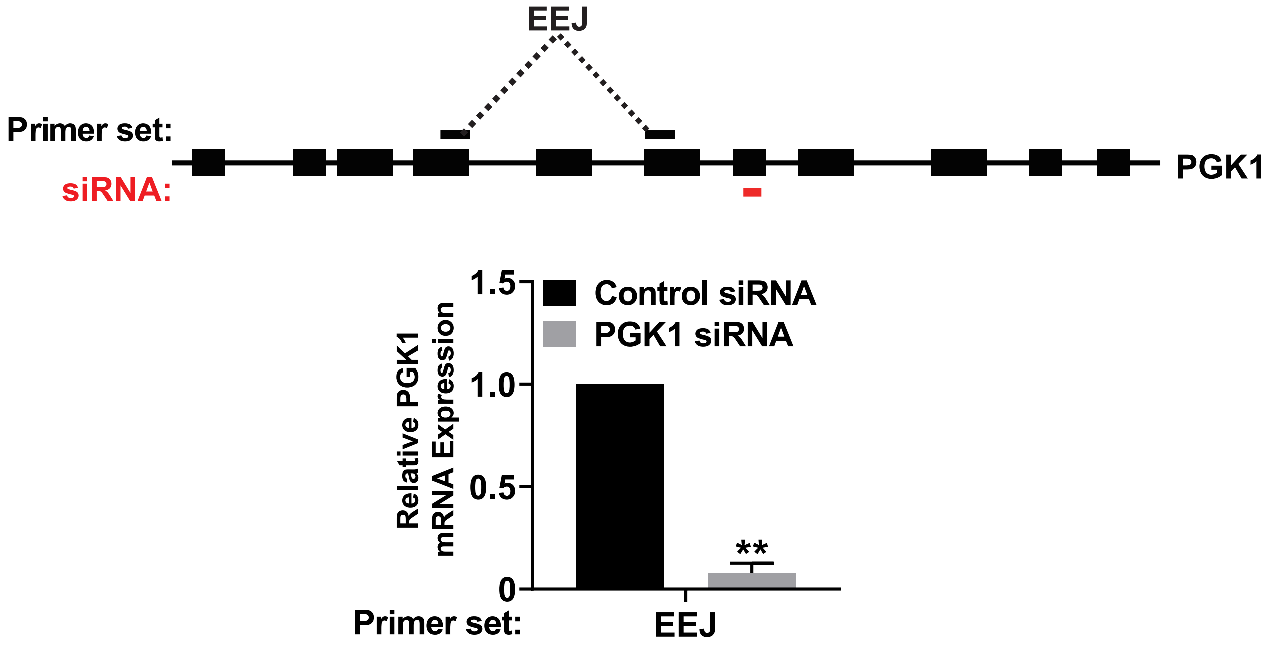

EEJ
Primer set:
siRNA:
PGK1
1.5
1.0
0.5
0
Control siRNA
PGK1 siRNA
Relative PGK1 mRNA Expression
**
Primer set:
EEJ

b

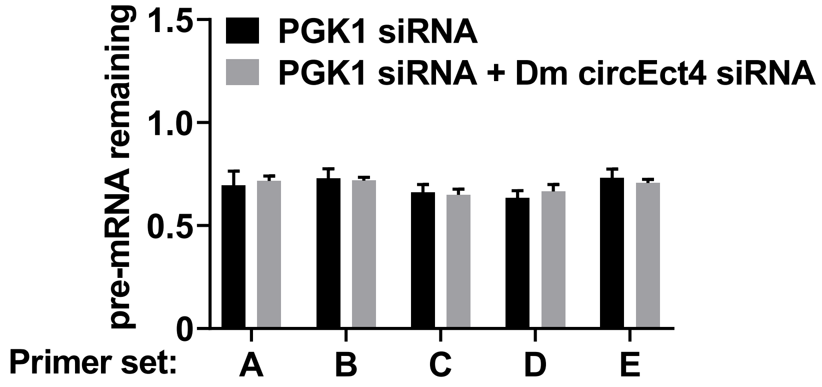

1.5
1.0
0.5
0
PGK1 siRNA
PGK1 siRNA + Dm circEct4 siRNA
pre-mRNA remaining
Primer set:
A
B
C
D
E

Figure S9

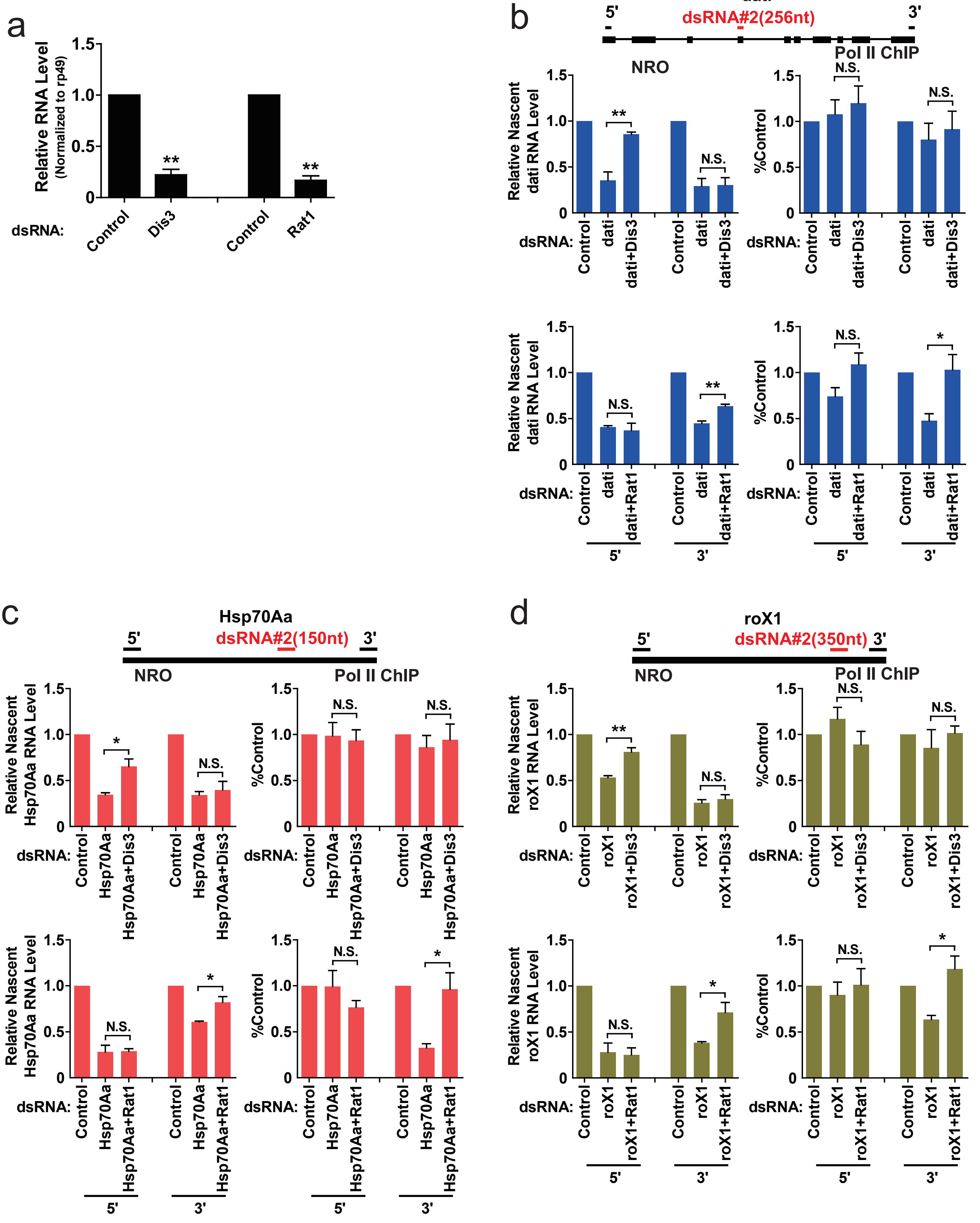
a
Relative RNA Level (Normalized to rp49)
dsRNA: Control Dis3 Control Rat1
b
dati
5'
dsRNA#2(256nt)
3'
NRO
Pol II ChIP
Relative Nascent dati RNA Level
%Control
dsRNA: Control dati dati+Dis3 Control dati dati+Dis3
dsRNA: Control dati dati+Rat1 Control dati dati+Rat1
5'
3'
N.S.
c
Hsp70Aa
dsRNA#2(150nt)
Relative Nascent Hsp70Aa RNA Level
dsRNA: Control Hsp70Aa Hsp70Aa+Dis3
dsRNA: Control Hsp70Aa Hsp70Aa+Rat1
d
roX1
dsRNA#2(350nt)
Relative Nascent roX1 RNA Level
dsRNA: Control roX1 roX1+Dis3
dsRNA: Control roX1 roX1+Rat1

Figure S10

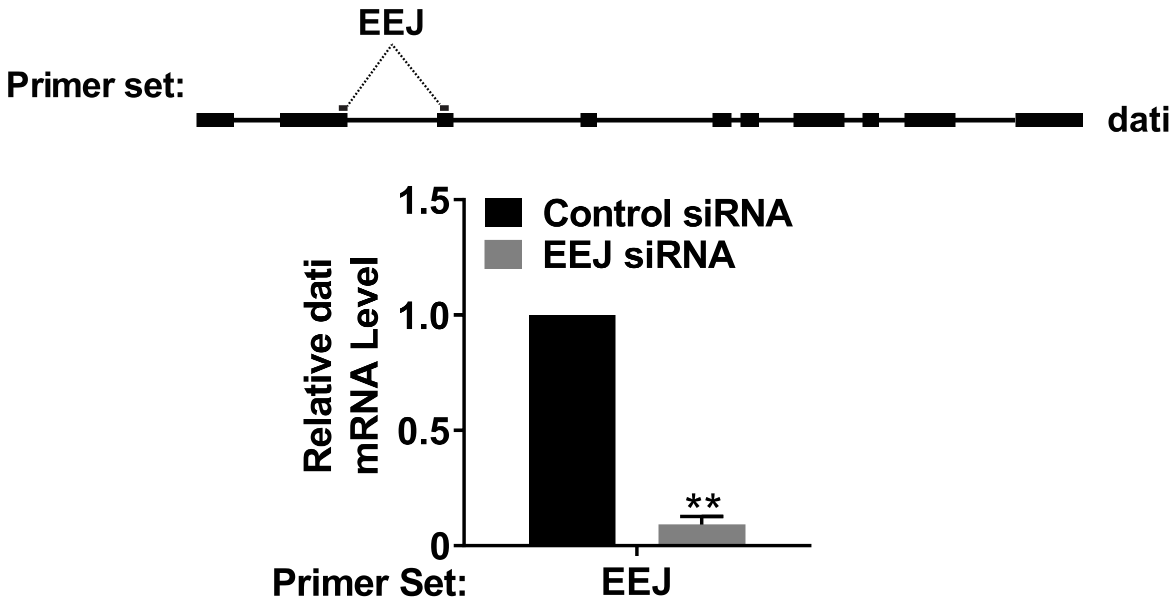
EEJ
Primer set:
dati
1.5
1.0
0.5
0
Control siRNA
EEJ siRNA
Relative dati
mRNA Level
**
Primer Set:
EEJ

SUPPLEMENTARY FIGURE LEGENDS

Figure S1. DsRNA efficiently reduces gene expression post-transcriptionally.

(a) Detailed information of four *Drosophila* genes that were selected for the following RNAi experiments.

(b) Real-time RT-PCR analysis of *MtnA*, *dati*, *Hsp70Aa*, or *roX1* level in RNA purified from S2 nuclear or cytoplasmic fraction. The enriched fraction is set to 1. Data are shown as mean ± SEM. n = 3.

(c-f) Real-time RT-PCR analysis of *MtnA* (**c**), *dati* (**d**), *Hsp70Aa* (**e**), or *roX1* (**f**) steady-state transcript level following dsRNA transfection. The indicated gene loci with the locations of PCR amplicons and dsRNA targeting sites are shown on top. Data were normalized to the control dsRNA sample and are shown as mean ± SEM. n = 3. ** $P < 0.01$; * $P < 0.05$.

(g, h) Real-time RT-PCR analysis of *MtnA* (**g**) or *dati* (**h**) pre-mRNA level following dsRNA transfection. Data were normalized to the control dsRNA sample and are shown as mean ± SEM. n = 3. ** $P < 0.01$; * $P < 0.05$.

Figure S2. Metabolic labeling experiments showing the effect of RNAi-mediated knockdown on production of nascent transcripts.

(a) Flow chart of the metabolic labeling experiment.

(b) Real-time RT-PCR analysis of *MtnA* (left) or *dati* (right) pre-mRNA enrichment in metabolic labeling sample over that in total RNA sample. Data were normalized to the total RNA sample and are shown as mean ± SEM. n = 3.

(c-e) Real-time RT-PCR analysis of *dati* (**c**), *Hsp70Aa* (**d**), or *roX1* (**e**) nascent transcript level in metabolic labeling experiments. The indicated gene loci with the locations of PCR amplicons and dsRNA targeting sites are shown on top. Data were normalized to the control dsRNA sample and are shown as mean ± SEM. n = 3. ** $P < 0.01$; * $P < 0.05$.

Figure S3. NRO experiments showing the effect of RNAi-mediated knockdown on production of nascent transcripts.

(a) Flow chart of the NRO experiment.

(b) Real-time RT-PCR analysis of *MtnA* (left) or *dati* (right) pre-mRNA enrichment in NRO sample over that in total RNA sample. Data were normalized to the total RNA sample and are shown as mean ± SEM. n = 3.

(c-e) Real-time RT-PCR analysis of *dati* (**c**), *Hsp70Aa* (**d**), or *roX1* (**e**) nascent transcript level in NRO experiments. The indicated gene loci with the locations of PCR amplicons and dsRNA targeting sites are shown on top. Data were normalized to the control dsRNA sample and are shown as mean ± SEM. n = 3. ** $P < 0.01$; * $P < 0.05$.

Figure S4. RNAi-mediated knockdown triggers premature transcription termination by releasing RNA Pol II from the region downstream of the dsRNA targeting site.

(a) Flow chart of the RNA Pol II ChIP experiment.

(b-e) Real-time ChIP-PCR analysis of RNA Pol II occupancy at the *dati* (**b**), *Hsp70Aa* (**c**), or *roX1* (**d**) locus. RNA Pol II occupancy at the *rp49* locus (**e**) served as a negative control. Pol II binding sites were defined as regions enriched over the input DNA. The indicated gene loci with the locations of PCR amplicons and dsRNA targeting sites are shown on top. Data were normalized to the control dsRNA sample and are shown as mean ± SEM. n = 3.

Figure S5. Ago2 is required for RNAi-induced nascent RNA degradation.

(a-c) Real-time RT-PCR analysis of *dati* (**a**), *Hsp70Aa* (**b**), or *roX1* (**c**) nascent transcript level in Ago2 knockdown or rescue experiments. The level of *rp49* nascent transcript served as a negative control. The indicated gene loci with the locations of PCR amplicons and dsRNA targeting sites are shown on top. Data were normalized to the control dsRNA sample and are shown as mean ± SEM. n = 3. ** $P < 0.01$; * $P < 0.05$.

(d) Real-time RT-PCR analysis of *MtnA* nascent transcript level in NRO sample from Ago2 knockout cells. Data were normalized to the control dsRNA sample and are shown as mean ± SEM. n = 3.

(e) Real-time ChIP-PCR analysis of RNA Pol II occupancy at the *MtnA* locus in Ago2 knockout cells. Data were normalized to the control dsRNA sample and are shown as mean ± SEM. n = 3.

Figure S6. Exogenous siRNAs partially co-localize with Ago2 in the nucleus and Ago2 depletion has little effect on the subcellular localization of exogenous siRNAs.

(a) Immunostaining analysis of the subcellular localization of Ago2 protein in S2 cells transfected with FAM-labeled *dati* siRNA. Representative images are shown. Scale bars, 5 μm.

(b) Subcellular localization analysis of FAM-labeled *dati* siRNA in Ago2-depleted cells. Representative images are shown. Scale bars, 15 μm.

(c) Statistics of FAM-labeled siRNA signal in the nucleus and cytoplasm. n = 29 cells for calculation.

Figure S7. DsRNA transfection promotes Ago2 nuclear translocation.

(a) Immunostaining analysis of the subcellular localization of Ago2 protein upon dsRNA treatment in S2 cells. Representative images are shown. Scale bars, 15 μm.

(b) Statistics of nuclear Ago2 signal. n = 30 cells for calculation.

Figure S8. *PGK1* siRNA efficiently reduces the level of *PGK1* mature mRNA.

(a) Real-time RT-PCR analysis of *PGK1* mature mRNA level in RNA purified from *PGK1* siRNA treated HeLa cells. The *PGK1* locus with the location of EEJ PCR amplicon is shown on top. Data were normalized to the control siRNA sample and are shown as mean ± SEM. n = 3. ** $P < 0.01$; * $P < 0.05$.

(b) Related to figure 1m, n, the control experiment (*PGK1* siRNA + siRNA targeting the unrelated RNA *Drosophila circEct4*) was performed to exclude the possibility that the adding of *Ago2* siRNA or *IMP8* siRNA makes *PGK1* siRNAs less effective simply due to the occupancy of RNAi machinery. Data were normalized to the control siRNA sample and are shown as mean ± SEM. n = 3. ** $P < 0.01$; * $P < 0.05$.

Figure S9. Dis3 and Rat1 are required for RNAi-induced nascent RNA degradation.

(a) Real-time RT-PCR analysis of *Dis3* or *Rat1* level to verify that each dsRNA efficiently depleted its targeted gene. Data were normalized to the control dsRNA sample and are shown as mean ± SEM. n = 3. ** $P < 0.01$; * $P < 0.05$.

(b-d) Real-time RT-PCR analysis of *dati* (**b**), *Hsp70Aa* (**c**), or *roX1*(**d**) nascent transcript level in Dis3 or Rat1 knockdown experiments (left). Real-time ChIP-PCR analysis of RNA Pol II occupancy at the *dati* (**b**), *Hsp70Aa* (**c**), or *roX1*(**d**) locus in Dis3 or Rat1 knockdown experiments (right). The indicated gene loci with the locations of PCR amplicons and dsRNA targeting sites are shown on top. Data were normalized to the control dsRNA sample and are shown as mean ± SEM. n = 3. ** $P < 0.01$; * $P < 0.05$.

Figure S10. Related to figure 1s, EEJ siRNA efficiently reduces the level of mature mRNA.

Real-time RT-PCR analysis of *dati* mature mRNA level in RNA purified from EEJ siRNA treated S2 cells. The *dati* locus with the location of EEJ PCR amplicon is shown on top. Data were normalized to the control siRNA sample and are shown as mean ± SEM. n = 3. ** $P < 0.01$; * $P < 0.05$.

SUPPLEMENTARY METHODS

Drosophila cell culture and RNAi

Drosophila Schneider 2 (S2) cells were cultured at 25 °C with Schneider's *Drosophila* medium (Sigma, S9895) plus 10% fetal bovine serum (HyClone, SH30910.03) and 1% (v/v) penicillin-streptomycin (Thermo Fisher Scientific, 15140122). DsRNAs were generated by in *vitro* transcription of PCR templates containing the T7 promoter sequence on both ends using ScriptMAX thermo T7 Transcription Kit (TOYOBO, TSK101). For dsRNA transfection, 3 million S2 cells were treated with 8 μg of the indicated dsRNA for 2 days. For siRNA transfection, 1.5 million S2 cells were transfected with the indicated siRNA (final concentration: 50 nM) for 2 days using Lipofectamine RNAiMAX (Thermo Fisher Scientific, 13778075) according to the manufacturer's instructions. Details for dsRNAs and siRNAs targeting *Drosophila* genes are provided in Supplementary Table S1 and S2.

Mammalian cell culture and RNAi

HeLa cells were cultured at 37 °C and 5% CO_2 with Minimum Essential Medium (Thermo Fisher Scientific, C11095500BT) plus 10% fetal bovine serum (Natocor - Industria Biológica, SFBE) and 1% (v/v) penicillin-streptomycin. siRNAs (final concentration: 100 nM) were introduced into HeLa cells for 2 days using Lipofectamine RNAiMAX. Details for siRNAs targeting human genes are provided in Supplementary Table S2.

Plasmids and plasmid transfection

To generate plasmids expressing flag-tagged Ago2 (WT and MUT), the indicated sequences were inserted into a pMK33/pMtHy-based vector (https://www.addgene.org/69911/) as previously described[1-3]. Cloning details for these plasmids are provided in Supplementary Plasmid Information.

To generate plasmids for construction of knockout cell line, sgRNA target sequences were designed by CRISPOR (http://crispor.tefor.net/). Sense and antisense

oligos were phosphorylated with T4 Polynucleotide Kinase (NEB, M0201S), annealed by slowly cooling from 95 °C to 25 °C, and inserted into the *Bsp* QI site of the pAc-sgRNA-Cas9 vector (a gift from Dr. Qingfa Wu, USTC).

Guide sequences are provided below:

5'- AGCTGTATGGTCCTGCAGG -3' and

5'- TTCAAATCCAGGAACCTGT -3' for genetic knockout of *Ago2* CDS in *Drosophila* genome;

For plasmid transfection, 3 million S2 cells were transfected with 2 µg of plasmid using Effectene transfection reagent (QIAGEN, 301425) according to the manufacturer's instructions.

Knockout cell construction

Knockout cell line was constructed following the protocol from Zhang *et al*. 2020[4]. In brief, plasmids encoding sgRNAs were introduced into S2 cells for 2 days using Effectene transfection reagent. A final concentration of 10 µg/ml puromycin (Beyotime, ST551) was added for another 3 days. Each cell clone was picked by a pipette tip and cultured for 3 weeks with fresh medium.

Chromatin immunoprecipitation (ChIP)

ChIP experiments were performed using ChIP Assay Kit (Beyotime, P2078) according to the manufacturer's instructions. In short, cells were fixed with formaldehyde, and sonicated in SDS lysis buffer to obtain chromatin solution containing 400-800 bp DNA fragments. Chromatin solution was precleared, and immunoprecipitated with an antibody against RNA Pol II (1:200 dilution; Abcam, ab5095) for 8 hours. Eluted DNA was then subjected to real-time PCR analysis to examine the enriched genomic DNA regions. Pol II binding sites were defined as regions enriched over the input DNA.

Cross-linking immunoprecipitation (CLIP)

CLIP was performed following the protocol from Li *et al.* 2015[5]. S2 cells were transfected with a transgene encoding wild-type or catalytically dead flag-tagged

Ago2 using Effectene transfection reagent on day 1, treated with the indicated dsRNAs on day 2, and collected for flag-IP after UV cross-linking (254 nm, 400 mJ/cm^2, 1 min) on day 4. Real-time RT-PCR was then performed with primer set B to measure *MtnA* pre-mRNA enrichment in flag-tagged Ago2 CLIP samples. Anti-FLAG (Beyotime, AF519, 1:100 dilution) was used in this experiment.

Metabolic labeling of nascent transcripts

Metabolic labeling of nascent RNAs and nascent RNA purification were performed as previously described[1, 2, 6]. In brief, S2 cells were treated with 250 μM 4-thiouridine (4sU) for 10 minutes. 50 μg of total RNA was biotinylated with 10 μg/mL MTSEA biotin-XX (Biotium, 90066; dissolved in dimethylformamide), and 4sU-labelled RNA was separated from total RNA using Dynabeads™ MyOne™ Streptavidin T1 beads (Thermo Fisher Scientific, 65602). Eluted RNA was then subjected to real-time RT-PCR analysis to examine the level of the nascent transcript in intact cell. To confirm that there was not significant variation across the biotinylation reactions, 2 ng of synthetic RNA was included in each reaction. Synthetic RNA sequence and related primer sets are provided in Supplementary Table S3.

Nuclear run-on (NRO)

Nuclear run-on experiments were performed as previously described, with modifications[5]. In brief, nuclei were isolated from S2 cells, and incubated with NTP mixture (10 mM ATP, GTP, CTP and BrUTP) for 10 minutes. BrUTP-labelled RNA was separated from 20 μg of nuclear RNA using anti-BrdU (Abcam, ab1893) and Dynabeads™ Protein G beads (Thermo Fisher Scientific, 10003D) according to the manufacturer's instructions. Eluted RNA was then subjected to real-time RT-PCR analysis to examine the level of the nascent transcript in isolated nuclei.

Reverse transcription and PCR reactions

RNA was purified using Trizol reagent (Thermo Fisher Scientific, 15596018) according to the manufacturer's instructions. RNA was reverse-transcribed to the complementary DNA (cDNA) using PrimeScript RT Master Mix (Takara, RR036A)

according to the manufacturer's instructions. Real-time PCR experiments were performed using Master Qpcr Mix-SYBR (TSINGKE, TSE203). All primer sequences are provided in Supplementary Table S4.

Immunostaining

Immunostaining was performed as described previously[2, 5, 7]. In brief, S2 cells were seeded on a concanavalin A-coated coverslip, and incubated with an antibody against endogenous Ago2 (Abcam, ab5072, 1:200 dilution) for 8 hours at 4 °C followed by incubating with a fluorescence secondary antibody (Abcam, ab150077, 1:100 dilution or Beyotime, A0468, 1:100 dliution).

RNA pulldown

To examine whether exogenous siRNA directly interacts with the targeted pre-mRNA, S2 cells were transfected with biotin-labeled siRNA (Supplementary Table S2) targeting exon 2 of *MtnA* for 2 days. Pulldown of biotin-labeled RNAs was performed as described previously, with minor modifications[5]. The siRNA-binding pre-mRNA was then examined by RT-PCR experiments.

Confocal microscopy and image quantification

Fluorescence signals were obtained by Leica TCS SP8 microscopy system and quantified by ImageJ software.

Nuclear and cytoplasmic fractionation

Cellular fractionation for S2 cells was performed as described previously[2]. Protein purified from nuclear or cytoplasmic fraction was examined by western blotting to detect the subcellular localization of Ago2. RNA purified from nuclear or cytoplasmic fraction was examined by real-time RT-PCR to detect the subcellular localization of *MtnA*, *dati*, *Hsp70Aa*, or *roX1* RNA.

Western blotting

Protein samples from nuclei, cytoplasm, whole cell or immunoprecipitation samples

were prepared using RIPA buffer (50mM Tris-HCl pH 7.4, 150mM NaCl, 0.1% (w/v) SDS, 1% (w/v) sodium deoxycholate, 1% (v/v) Triton X-100). Protein samples were separated and detected as described previously[1, 2, 7]. These antibodies were used in western blots: anti-α-Tubulin (Beyotime, AT819, 1:1000 dilution), anti-HDAC1 (Abcam, ab1767, 1:1000 dilution), anti-RNA Pol II (Abcam, ab5095, 1:2000 dilution), anti-Histone H3 (Abcam, ab1791, 1:1000 dilution) and anti-Ago2 (Abcam, ab5072, 1:1000 dilution). Blots were viewed with a Bio-Rad ChemiDoc imaging system.

Statistical analyses

Statistical significance for comparisons of means was assessed by Student's t-test. Statistical details and error bars are defined in each figure legend: $P< 0.01$ (**) and $P <0.05$ (*)

SUPPLEMENTARY REFERENCES

1 Huang C, Liang D, Tatomer DC, Wilusz JE. A length-dependent evolutionarily conserved pathway controls nuclear export of circular RNAs. *Genes Dev* 2018; **32**:639-644.

2 Jia R, Xiao MS, Li Z, Shan G, Huang C. Defining an evolutionarily conserved role of GW182 in circular RNA degradation. *Cell Discov* 2019; **5**:45.

3 Tatomer DC, Elrod ND, Liang D *et al.* The Integrator complex cleaves nascent mRNAs to attenuate transcription. *Genes Dev* 2019; **33**:1525-1538.

4 Zhang L, Xu W, Gao X *et al.* lncRNA Sensing of a Viral Suppressor of RNAi Activates Non-canonical Innate Immune Signaling in Drosophila. *Cell Host Microbe* 2020; **27**:115-128 e118.

5 Li Z, Huang C, Bao C *et al.* Exon-intron circular RNAs regulate transcription in the nucleus. *Nat Struct Mol Biol* 2015; **22**:256-264.

6 Song Z, Jia R, Tang M *et al.* Antisense oligonucleotide technology can be used to investigate a circular but not linear RNA-mediated function for its encoded gene locus. *Sci China Life Sci* 2021; **64**:784-794

7 Huang C, Wang X, Liu X, Cao S, Shan G. RNAi pathway participates in chromosome segregation in mammalian cells. *Cell Discov* 2015; **1**:15029.

SUPPLEMENTARY TABLES

Table S1

dsRNA Target	Forward Primer	Reverse Primer	DRSC ID
Control (β-gal)	TAATACGACTCACTATAGGG CTGGCGTAATAGCGAAGAGG	TAATACGACTCACTATAGGG CATTAAAGCGAGTGGCAACA	DRSC42733
Rat1	TAATACGACTCACTATAGGG AAGAAGTGCGTTTACAAGACAAGG	TAATACGACTCACTATAGGG GCCCTGATAGTAGTACTTAAGAACCC	DRSC02044
Dis3	TAATACGACTCACTATAGGG ATCATCGTAACGATTGACACA	TAATACGACTCACTATAGGG CTTCATTGTCCACTTCCCAC	DRSC16034
Ago2	TAATACGACTCACTATAGGG GCAACGTATTGAATCTTATTATTTTTTATCTTT	TAATACGACTCACTATAGGG GATTCAACCCATAAGATGAGCTTTT	N/A
Hsp70Aa#1	TAATACGACTCACTATAGGG GTGCTCCGCATCATCAATGAGC	TAATACGACTCACTATAGGG GAACAGTGATCCCTCGTCGATGG	N/A
Hsp70Aa#2	TAATACGACTCACTATAGGG GAGAGCTACGTCTTCAATGTGAAGC	TAATACGACTCACTATAGGG GTCGAACTCCTCCTTCTCGGC	N/A
MtnA	TAATACGACTCACTATAGGG AGGCCACCAAGGGATCCT	TAATACGACTCACTATAGGG CATCAATTACTTTAATTGGACATTT	DRSC21588
dati#1	TAATACGACTCACTATAGGG GCGAGCTGGCAAATCTGTAT	TAATACGACTCACTATAGGG TCGCTTCCATATGACTGCTG	DRSC25407
dati#2	TAATACGACTCACTATAGGG AGCAGCAGATTCCTCGCGT	TAATACGACTCACTATAGGG TTACCTGTGTGCAGGCGC	N/A
roX1#1	TAATACGACTCACTATAGGG GCAGTAAACGACTGCAAAAG	TAATACGACTCACTATAGGG AGACTGATTCATGAATGAATATTTT	DRSC21884
roX1#2	TAATACGACTCACTATAGGG AAGTAGATCGTGTTCTGTGAACTAACCC	TAATACGACTCACTATAGGG AACCGTAATGAATGCATAGGCTTTCAAT	N/A

Table S2

siRNA	Antisense Sequence	Target
Control siRNA	UUCUCCGAACGUGUCACGUUU	
PGK1 siRNA	UUGUCUGCAACUUUAGCUCCG	human PGK1
Ago2 siRNA	UUCUUUGGUAAACUUUACCCU	human Ago2
IMP8 siRNA (Weinmann et al. Cell, 2009)	UUAAUUGGACUCUCACUAAUU	human IMP8
Intron dati siRNA	GGUCUGUGCGACAGCUCACAAAUU	*Drosophila* dati
circdati siRNA	CAUUUUAUUGCCUGAAUUUCAUUU	*Drosophila* circdati
EEJ dati siRNA	GAUAGCUGAAGCUGAAUUUCAUU	*Drosophila* dati
5'-FAM labeled dati siRNA/Exon dati siRNA	CUAGGCGUAAUACUGCCACCUAAGUU	*Drosophila* dati
5'-biotin labeled MtnA siRNA	CUCACUCGGAGCAGCCGCAGG	*Drosophila* MtnA
circEct4 siRNA	GUCUGUCAUCUUAAUGACGGCUGC	*Drosophila* circEct4

Table S3

For Metabolic labeling of nascent RNA	
Synthetic RNA sequence	5'-AUUUAGGUGACACUAUAGGAUCCUCUAGAGUCGACCUUCUCCCUAUAGUGAGUCGUAUUAGCA[4-S-U]CAG-3'
qPCR_F	ATTTAGGTGACACTATAGGATCCTCTAG
qPCR_R	GCTAATACGACTCACTATAGGGAGAAG

Table S4

PCR Target	Forward Primer	Reverse Primer	Note
DmHsp70Aa ChIP-PCR#A	TCAATTCTATTCAAACAAGTAAAGTGAACACA	GCACTTTATTGCAGATTGTTTAGCTTGT	also used for RT-PCR#A, 5'
DmHsp70Aa ChIP-PCR#B	GTTGTAAGCGACGGCGGAAAG	TCTTGGTCAGTACCATCGAGCT	also used for RT-PCR#B
DmHsp70Aa ChIP-PCR#C	TTCTACACCAAAGTGAGCCGC	TCTTGGCATCGTTGAGGGC	also used for RT-PCR#C
DmHsp70Aa ChIP-PCR#D	TATGAGGGCGAACGTGCGAT	CTTCTATCTGGGGCACACCC	also used for RT-PCR#D
DmHsp70Aa ChIP-PCR#E	GTCGACTAAGGCCAAAGAGTCTAATT	CGATCGAAACATTCTTATCAGTCTCAAAA	also used for RT-PCR#E, 3'
DmMtnA ChIP-PCR#A	TGCATCAGTTGTGGTCAGCAG	CTTGTTCACTTGTTTACAAAAAAGGTAGGT	also used for RT-PCR#A, 5'
DmMtnA ChIP-PCR#B	GTAAGTTCGCAGTCTGGTGTGAT	TTCACTTTGCAGATAGTTTCAGCTTG	also used for RT-PCR#B, 5'
DmMtnA ChIP-PCR#C	GGGTTGCATAATCAATTGGTCTGC	CCATTTCCACGACGCATATTGCC	also used for RT-PCR#C
DmMtnA ChIP-PCR#D	GCCTCACTTCTGAAGCGACTGA	TGATAGGGTAATATATTTTTATTATTTCTAAAGCTAGTAT	also used for RT-PCR#D, 3'
DmMtnA PCR#EEJ	GTGAACAAGTTCGAGGAAATACAACTCA	CCGCAGGCGGATTTCTTGTC	
Dmdati ChIP-PCR#A	GTTATCTGTAGTTGTTTGCTGGGAGC	CTCTTTACGAGGTTTTTTTTGTTGGAAGAA	also used for RT-PCR#A, 5'
Dmdati ChIP-PCR#B	CCTGAGGCACTAGAGAAATGTTGT	GGACAAGAAAAGACTGCTTTGGC	
Dmdati ChIP-PCR#C	CAGACGGATATGGCCAGTTCGA	CCCATTACTCGTAGAGGGAAAGTGTA	
Dmdati ChIP-PCR#D	CTTCAGCTATCCCGTTCGAGC	TCGTTGTAGGGTTGACCAACATT	also used for RT-PCR#B
Dmdati ChIP-PCR#E	GAACGACCCTATAAATGCCATCTGC	ACGCAGGCTAGATTCAGTGG	also used for RT-PCR#C
Dmdati ChIP-PCR#F	ACGTACTTAACTGAACTTAACTTAAAGATAACT	TGAGCAGAAAAGGACAAGACAAAACG	also used for RT-PCR#D
Dmdati ChIP-PCR#G	GGCAGTCATGCCCTAAGTTAGAA	AATTTTCGAGCATTTATCTCTAAACTTATTATAAACGT	also used for RT-PCR#E, 3'
Dmdati PCR#EEJ	CGACGCCCGACATCAAATACA	AACATTCTTCGAGGGTGCGT	
Dmcircdati PCR	GGTGCCAACTGTGCGAAGTT	CGACGCCCGACATCAAATACAAT	
DmroX1 ChIP-PCR#A	GATCATCAGTCTTTGGCGGCATG	CAGTAGCAGTACACACTCTAGCTAA	also used for RT-PCR#A, 5'
DmroX1 ChIP-PCR#B	TGAACAAGTACTCAGAAATCGCTCGA	CAGTTAATAAACTGATTAATTTCTGTAAGCAATATTTTGG	also used for RT-PCR#B
DmroX1 ChIP-PCR#C	CCTTACACCGGGCACCTACAAT	CTTTGGCTCCGCGAGGCT	also used for RT-PCR#C
DmroX1 ChIP-PCR#D	GATATTTACAAACGGGGTTATCTCTATAAGG	GATATTTATAAAAATAGTGATAGGGATAGTTAGGTATGT	also used for RT-PCR#D, 3'
Dmrp49 ChIP-PCR#A	CCATTAATTTTAAGCCGTAATTGTCGTTTT	CCGAATTCGGTCGAAATACGAGC	
Dmrp49 ChIP-PCR#B	AAGATGACCATCCGCCCAG	CGACAGCTTAGCATATCGATCCG	also used for RT-PCR
Dmrp49 ChIP-PCR#C	GCCCAAGGGTATCGACAACAGA	GCAGCATGTGGCGGGTG	
Dmrp49 ChIP-PCR#D	CGGTCCGCCTCACCAAC	GTGTATTCCGACCACGTTACAAGAA	
DmRat1 PCR	CCGTCTGTTCGGTATTGTACG	GAGATCGCTGCTGATTCATCTT	
DmDis3 PCR	AACGAGGTGAAGCACAGGAG	GGCGGTCTCATCTGGTTCG	
DmAgo2 PCR	CAGAGCCAAGGCCAATACCA	AGTTGATGCCTACTTGCCCG	
human-PGK1 PCR#A	TATCTGCCAGAATTGTTGGTAGAGTG	ATCAGTTACATACCGACTCATCCTC	
human-PGK1 PCR#B	CTGAATATTCGAGACAATGACCAAGG	GGCCTACAATGTGACTCTCTGTTC	
human-PGK1 PCR#C	GCAGCCATAGATAACTCACCAAAC	TCCGAGATACTAAAGACCAGAATAGTG	
human-PGK1 PCR#D	CTTGTAGACTACCACACTGAGAACA	CAACAAGAGCCATAGTGGCAGT	
human-PGK1 PCR#E	GCAGTGCTATTGGATTACTGGC	CCTGGTGACACAGCGAGACT	
human-PGK1 PCR#EEJ	TCTGTCATCCTGCTGGAGAAC	GTGGCAGATTGACTCCTACCAT	
human-Ago2 PCR	AGGATATGCCTTCAAGCCTCC	GGCACTTCTCTGGCTTGATATC	
human-IMP8 PCR	AGCAGCCCAGACTCTCTTATATAC	CTCGAAGATATCCCAGGTTAGACA	

SUPPLEMENTARY PLASMID INFORMATION

*There were **6** plasmids used in this study:*

1. **Hy_pMtnA EGFP SV40 pA Sense** (original plasmid, used for subsequent cloning, see Page 2)
2. **Hy_pMtnA Flag MCS** (used for subsequent cloning, see Page 5)
3. **Hy_MCS Flag MCS** (used for subsequent cloning, see Page 5)
4. **Hy_pAct5C Flag MCS** (used for subsequent cloning, see Page 5)
5. **Hy_pAct5C Flag_Ago2_WT** (used for Figure 3 and S4, see Page 6)
6. **Hy_pAct5C Flag_Ago2_MUT** (used for Figure 3 and S4, see Page 7)

All expression plasmids of *Drosophila* were generated from the **<u>Hy_pMtnA EGFP SV40 pA Sense</u>** plasmid (https://www.addgene.org/69911/), which is a modified form of the pMK33/pMtHy plasmid. Briefly, expression of the EGFP ORF (marked in green) that terminates in the SV40 polyadenylation signal (marked in pink) is driven by the metallothionein A (MtnA) promoter (marked in blue). The copia transposon LTR promoter drives HygroR (marked in red) that terminates with an SV40 late poly(A) signal (marked in gray). An Amp selectable marker is also present. The full plasmid sequence is as follows:

GATCAATTCGTTGCAGGACAGGATGTGGTGCCCGATGTGACTAGCTCTTTGCTGCAGGCCGTCCTATCCTCTGGTTCCGAT
AAGAGACCCAGAACTCCGGCCCCCCACCGCCCACCGCCACCCCCATACATATGTGGTACGCAAGTAAGAGTGCCTGCGCAT
GCCCCATGTGCCCCACCAAGAGCTTTGCATCCCATACAAGTCCCCAAAGTGGAGAACCGAACCAATTCTTCGCGGGCAGAA
CAAAAGCTTCTGCACACGTCTCCACTCGAATTTGGAGCCGGCCGGCGTGTGCAAAAGAGGTGAATCGAACGAAAGACCCGT
GTGTAAAGCCGCGTTTCCAAAATGTATAAAACCGAGAGCATCTGGCCAATGTGCATCAGTTGTGGTCAGCAGCAAAATCAA
GTGAATCATCTCAGTGCAACTAAAGGGGGGATCTCGAGGTCGACGGTATCGATAAGCTTGATATCACCATGGTGAGCAAGG
GCGAGGAGCTGTTCACCGGGGTGGTGCCCATCCTGGTCGAGCTGGACGGCGACGTAAACGGCCACAAGTTCAGCGTGTCCG
GCGAGGGCGAGGGCGATGCCACCTACGGCAAGCTGACCCTGAAGTTCATCTGCACCACCGGCAAGCTGCCCGTGCCCTGGC
CCACCCTCGTGACCACCCTGACCTACGGCGTGCAGTGCTTCAGCCGCTACCCCGACCACATGAAGCAGCACGACTTCTTCA
AGTCCGCCATGCCCGAAGGCTACGTCCAGGAGCGCACCATCTTCTTCAAGGACGACGGCAACTACAAGACCCGCGCCGAGG
TGAAGTTCGAGGGCGACACCCTGGTGAACCGCATCGAGCTGAAGGGCATCGACTTCAAGGAGGACGGCAACATCCTGGGGC
ACAAGCTGGAGTACAACTACAACAGCCACAACGTCTATATCATGGCCGACAAGCAGAAGAACGGCATCAAGGTGAACTTCA
AGATCCGCCACAACATCGAGGACGGCAGCGTGCAGCTCGCCGACCACTACCAGCAGAACACCCCCATCGGCGACGGCCCCG
TGCTGCTGCCCGACAACCACTACCTGAGCACCCAGTCCGCCCTGAGCAAAGACCCCAACGAGAAGCGCGATCACATGGTCC
TGCTGGAGTTCGTGACCGCCGCCGGGATCACTCTCGGCATGGACGAGCTGTACAAGTAAGCGGCCGCAACTTGTTTATTGC
AGCTTATAATGGTTACAAATAAAGCAATAGCATCACAAATTTCACAAATAAAGCATTTTTTTCACTGCATTCTAGTTGTGG
TTTGTCCAAACTCATCAATGTATCTTAACTAGTGGATCCACTAGGGGCCGCCGACGCGAGGCTGGATGGCCTTCCCCATTA
TGATTCTTCTCGCTTCCGGCGGCATCGGGATGCCCGCGTTGCAGGCCATGCTGTCCAGGCAGGTAGATGACGACCATCAGG
GACAGCTTCAAGGATCGCTCGCGGCTCTTACCAGCCTAACTTCGATCATTGGACCGCTGATCGTCACGGCGATTTATGCCG
CCTCGGCGAGCACATGGAACGGGTTGGCATGGATTGTAGGCGCCGCCCTATACCTTGTCTGCCTCCCCGCGTTGCGTCGCG
GTGCATGGAGCCGGGCCACCTCGACCTGAATGGAAGCCGGCGGCACCTCGCTAACGGATTCACCACTCCAAGAATTGGAGC
CAATCAATTCTTGCGGAGAACTGTGAATGCGCAAACCAACCCTTGGCAGAACATATCCATCGCGTCCGCCATCTCCAGCAG
CCGCACGCGGCGCATCTCGGGCAGCGTTGGGTCCTGGCCACGGGTGCGCATGATCGTGCTCCTGTCGTTGAGGACCCGGCT
AGGCTGGCGGGGTTGCCTTACTGGTTAGCAGAATGAATCACCGATACGCGAGCGAACGTGAAGCGACTGCTGCTGCAAAAC
GTCTGCGACCTGAGCAACAACATGAATGGTCTTCGGTTTCCGTGTTTCGTAAAGTCTGGAAACGCGGAAGTCAGCGCTCTT
CCGCTTCCTCGCTCACTGACTCGCTGCGCTCGGTCGTTCGGCTGCGGCGAGCGGTATCAGCTCACTCAAAGGCGGTAATAC
GGTTATCCACAGAATCAGGGGATAACGCAGGAAAGAACATGTGAGCAAAAGGCCAGCAAAAGGCCAGGAACCGTAAAAAGG
CCGCGTTGCTGGCGTTTTTCCATAGGCTCCGCCCCCCTGACGAGCATCACAAAAATCGACGCTCAAGTCAGAGGTGGCGAA
ACCCGACAGGACTATAAAGATACCAGGCGTTTCCCCCTGGAAGCTCCCTCGTGCGCTCTCCTGTTCCGACCCTGCCGCTTA
CCGGATACCTGTCCGCCTTTCTCCCTTCGGGAAGCGTGGCGCTTTCTCATAGCTCACGCTGTAGGTATCTCAGTTCGGTGT
AGGTCGTTCGCTCCAAGCTGGGCTGTGTGCACGAACCCCCCGTTCAGCCCGACCGCTGCGCCTTATCCGGTAACTATCGTC
TTGAGTCCAACCCGGTAAGACACGACTTATCGCCACTGGCAGCAGCCACTGGTAACAGGATTAGCAGAGCGAGGTATGTAG
GCGGTGCTACAGAGTTCTTGAAGTGGTGGCCTAACTACGGCTACACTAGAAGGACAGTATTTGGTATCTGCGCTCTGCTGA
AGCCAGTTACCTTCGGAAAAAGAGTTGGTAGCTCTTGATCCGGCAAACAAACCACCGCTGGTAGCGGTGGTTTTTTTGTTT

GCAAGCAGCAGATTACGCGCAGAAAAAAAGGATCTCAAGAAGATCCTTTGATCTTTTCTACGGGGTCTGACGCTCAGTGGA
ACGAAAACTCACGTTAAGGGATTTTGGTCATGAGATTATCAAAAAGGATCTTCACCTAGATCCTTTTAAATTAAAAATGAA
GTTTTAAATCAATCTAAAGTATATATGAGTAAACTTGGTCTGACAGTTACCAATGCTTAATCAGTGAGGCACCTATCTCAG
CGATCTGTCTATTTCGTTCATCCATAGTTGCCTGACTCCCCGTCGTGTAGATAACTACGATACGGGAGGGCTTACCATCTG
GCCCCAGTGCTGCAATGATACCGCGAGACCCACGCTCACCGGCTCCAGATTTATCAGCAATAAACCAGCCAGCCGGAAGGG
CCGAGCGCAGAAGTGGTCCTGCAACTTTATCCGCCTCCATCCAGTCTATTAATTGTTGCCGGGAAGCTAGAGTAAGTAGTT
CGCCAGTTAATAGTTTGCGCAACGTTGTTGCCATTGCTGCAGGCATCGTGGTGTCACGCTCGTCGTTTGGTATGGCTTCAT
TCAGCTCCGGTTCCCAACGATCAAGGCGAGTTACATGATCCCCCATGTTGTGCAAAAAAGCGGTTAGCTCCTTCGGTCCTC
CGATCGTTGTCAGAAGTAAGTTGGCCGCAGTGTTATCACTCATGGTTATGGCAGCACTGCATAATTCTCTTACTGTCATGC
CATCCGTAAGATGCTTTTCTGTGACTGGTGAGTACTCAACCAAGTCATTCTGAGAATAGTGTATGCGGCGACCGAGTTGCT
CTTGCCCGGCGTCAACACGGGATAATACCGCGCCACATAGCAGAACTTTAAAAGTGCTCATCATTGGAAAACGTTCTTCGG
GGCGAAAACTCTCAAGGATCTTACCGCTGTTGAGATCCAGTTCGATGTAACCCACTCGTGCACCCAACTGATCTTCAGCAT
CTTTTACTTTCACCAGCGTTTCTGGGTGAGCAAAAACAGGAAGGCAAAATGCCGCAAAAAAGGGAATAAGGGCGACACGGA
AATGTTGAATACTCATACTCTTCCTTTTTCAATATTATTGAAGCATTTATCAGGGTTATTGTCTCATGAGCGGATACATAT
TTGAATGTATTTAGAAAAATAAACAAATAGGGGTTCCGCGCACATTTCCCCGAAAAGTGCCACCTGACGTCTAAGAAACCA
TTATTATCATGACATTAACCTATAAAAATAGGCGTATCACGAGGCCCTTTCGTCTTCAAGAATTCACATTTGTACGAATTT
TTTTTTTATCAAAAGTTCGAGTTTTTCACCAATTTCCTCATCAACCGAGCAAGGCAAACGGCTTTGAATAATATGGTGTTA
TATATACATATATCAAATCGCTGCTGACTGCGTGATTGATGGCCCCAAGATTACATATTATCGAATCAGGATTCAGAAGGA
GATCAATGTCAAATGCGGACAGGAACATGAAAGACGCCTGTTATGCGCAATTAAAAATTTGGGTTTAATTGCTGTGGAAAC
TGTTGTTGGCGGCATCTTAAGTTCCTGTTTAACAACATCAACTACTTATGTACGTAGAAGCGTTTAAGCCATTTGCATACA
GATGAGAACTGGCTTTTGTGCTAATCAGTCAAGATGACTCCGATGATGATGACTCATTACCTGACCAGTTTTCGCTGCTTT
CTTTTCAACAACTACTTGTATATGTATTGTATCCAATAGCAATACATTGAATTTCCATGGTCTAGTCACGTATTATCATTT
AATTGACACCAAGTCGTGTTATTGTTGAGCTATCGAGTTCAGCTCAAACATTTCTTATTCCCATGAATAAGCCGGCAAAAA
TATGCAATCTATGAAAGTTAATATAAGCAAACCTTACTTTGACTCAATACCAATGCACTTTGTGTCGATAGGTTCACGCAA
TTGAGGCGATTATTCCGATAACCCAAGCGATTGACTGTTCCCGTTTCGATTCCAATTGAAATTTGGAAATGTACAATAGTT
TTGCTATATGCTGTCAAGTACGCTCTTATCTTCTCTGGGTTTTCTTCAGAGTTTCGAAACGCTTCTTCTTTTTTTTTGTTTT
TTTTTTTTTGGAATCTCGTATTTTGGAAGGGGCTCCCCTCTGGAATTTGTTACACTGTCGTTATCATTGCGAACAAGCGGC
CCGAAGCTATCAGCGACTTTAACATTTACAATGCACTTTTTTACGACCAATTAAATGTACATTTTCCTTTCTTCGCCCGTT
GATAAGCGAACGCGATGTGGCGCAGGCAATGTGTTGCTCTTGCGACACAAACGCAATCAAAATGGATTCAATTTCGCTTTT
TCCCAGTGAAACGAAGAACGAACCGACCATCATGATATGCTCCTCTGCATGTTGCGTATTGAATCAATGACAATTTCAATT
AAGCCGCCCGTTCGTCATGCGTTTTCGTGCGCTTCGAAATGCTGATAACGCTGCTGTCCTCCAACTGCTTTGCATGTGGAC
ACAATTCCATTTATTTAATTCTTTTATTTGGATCGGTTAAATTAAAAAGCGCCTTGTTACGCATTTAACGTTGTTTCCGGT
GCGTGGTGGTTTCATGCTTCTGGGAACGGCAAATGGGTTTAGGATTGGGAACCCCTCATCATCTGTTGGAATATACTATTC
AACCTACAAAAGTAACGTTAAACAACACTACTTTATATTTGATATGAATGGCCACACCTTTTATGCCATAAAACATATTGT
AAGAGAATACCACTCTTTTTATTCCTTCTTTCCTTCTTGTACGTTTTTTGCTGTAAGTAGGTCGTGGTGCTGGTGTTGCAG
TTGAAATAACTTAAAATATAAATCATAAAACTCAAACATAAACTTGACTATTTATTTATTTATTAAGAAAGGAAATATAAA
TTATAAATTACAACAGGTTATGGACCTGCAGCCAAGCTTGGCGCTCGTCCGGGGGCAATGAGATATGAAAAAGCCTGAACT
CACCGCGACGTCTGTCGAGAAGTTTCTGATCGAAAAGTTCGACAGCGTCTCCGACCTGATGCAGCTCTCGGAGGGCGAAGA
ATCTCGTGCTTTCAGCTTCGATGTAGGAGGGCGTGGATATGTCCTGCGGGTAAATAGCTGCGCCGATGGTTTCTACAAAGA
TCGTTATGTTTATCGGCACTTTGCATCGGCCGCGCTCCCGATTCCGGAAGTGCTTGACATTGGGGAATTCAGCGAGAGCCT
GACCTATTGCATCTCCCGCCGTGCACAGGGTGTCACGTTGCAAGACCTGCCTGAAACCGAACTGCCCGCTGTTCTGCAGCC
GGTCGCGGAGGCCATGGATGCGATCGCTGCGGCCGATCTTAGCCAGACGAGCGGGTTCGGCCCATTCGGACCGCAAGGAAT
CGGTCAATACACTACATGGCGTGATTTCATATGCGCGATTGCTGATCCCCATGTGTATCACTGGCAAACTGTGATGGACGA
CACCGTCAGTGCGTCCGTCGCGCAGGCTCTCGATGAGCTGATGCTTTGGGCCGAGGACTGCCCCGAAGTCCGGCACCTCGT

GCACGCGGATTTCGGCTCCAACAATGTCCTGACGGACAATGGCCGCATAACAGCGGTCATTGACTGGAGCGAGGCGATGTT
CGGGGATTCCCAATACGAGGTCGCCAACATCTTCTTCTGGAGGCCGTGGTTGGCTTGTATGGAGCAGCAGACGCGCTACTT
CGAGCGGAGGCATCCGGAGCTTGCAGGATCGCCGCGGCTCCGGGCGTATATGCTCCGCATTGGTCTTGACCAACTCTATCA
GAGCTTGGTTGACGGCAATTTCGATGATGCAGCTTGGGCGCAGGGTCGATGCGACGCAATCGTCCGATCCGGAGCCGGGAC
TGTCGGGCGTACACAAATCGCCCGCAGAAGCGCGGCCGTCTGGACCGATGGCTGTGTAGAAGTACTCGCCGATAGTGGAAA
CCGACGCCCCAGCACTCGTCCGAGGGCAAAGGAATAGAGTAGATGCCGACCGAACAAGAGCTGATTTCGAGAACGCCTCAG
CCAGCAACTCGCGCGAGCCTAGCAAGGCAAATGCGAGAGAACGGCCTTACGCTTGGTGGCACAGTTCTCGTCCACAGTTCG
CTAAGCTCGCTCGGCTGGGTCGCGGGAGGGCCGGTCGCAGTGATTCAGGCCCTTCTGGATTGTGTTGGTCCCCAGGGCACG
ATTGTCATGCCCACGCACTCGGGTGATCTGACTGATCCCGCAGATTGGAGATCGCCGCCCGTGCCTGCCGATTGGGTGCAG
ATCTTTGTGAAGGAACCTTACTTCTGTGGTGTGACATAATTGGACAAACTACCTACAGAGATTTAAAGCTCTAAGGTAAAT
ATAAAATTTTTAAGTGTATAATGTGTTAAACTACTGATTCTAATTGTTTGTGTATTTTAGATTCCAACCTATGGAACTGAT
GAATGGGAGCAGTGGTGGAATGCCTTTAATGAGGAAAACCTGTTTTGCTCAGAAGAAATGCCATCTAGTGATGATGAGGCT
ACTGCTGACTCTCAACATTCTACTCCTCCAAAAAAGAAGAGAAAGGTAGAAGACCCCAAGGACTTTCCTTCAGAATTGCTA
AGTTTTTTGAGTCATGCTGTGTTTAGTAATAGAACTCTTGCTTGCTTTGCTATTTACACCACAAAGGAAAAAGCTGCACTG
CTATACAAGAAAATTATGGAAAAATATTCTGTAACCTTTATAAGTAGGCATAACAGTTATAATCATAACATACTGTTTTTT
CTTACTCCACACAGGCATAGAGTGTCTGCTATTAATAACTATGCTCAAAAATTGTGTACCTTTAGCTTTTTAATTTGTAAA
GGGGTTAATAAGGAATATTTGATGTATAGTGCCTTGACTAGAGATCATAATCAGCCATACCACATTTGTAGAGGTTTTACT
TGCTTTAAAAAACCTCCCACACCTCCCCCTGAACCTGAAACATAAAATGAATGCAATTGTTGTTGTTAACTTGTTTATTGC
AGCTTATAATGGTTACAAATAAAGCAATAGCATCACAAATTTCACAAATAAAGCATTTTTTTCACTGCATTCTAGTTGTGG
TTTGTCCAAACTCATCAATGTATCTTATCATGTCTG

The following plasmid was generated by inserting the indicated sequences into the XhoI and NotI sites of **Hy_pMtnA EGFP SV40 pA Sense**, thereby replacing the EGFP ORF with the indicated sequence.

<u>Hy_pMtnA Flag MCS</u>

Note: This plasmid was used for subsequent cloning

Previously described in Huang et al. 2018.

ACCATGGACTACAAAGACCATGACGGTGATTATAAAGATCATGACATCGATTACAAGGATGACGATGACAAGACCGGTGGT
ACCGCTAGCTTAATTAAGAGCTCCCCGGG

The following plasmid was generated by replacing the MtnA promoter of **Hy_pMtnA Flag MCS** with the indicated sequence (multiple clone site [MCS]).

<u>Hy_MCS Flag MCS</u>

Note: This plasmid was used for subsequent cloning

GGGCCCTCTAGAGATATCGTCGACAGGCCTACGCGT

The following plasmid was generated by inserting indicated sequence (β-actin promoter) into the XbaI and SalI sites of **Hy_MCS Flag MCS**.

<u>Hy_pAct5C Flag MCS</u>

AGATCTCGCTGCCTGTTATGTGGCCCACAAACCAAGACACGTTTTATGGCCATTAAAGCTGGCTGATCGTCGCCAAACACC
AAATACATAATGAATATGTACACATTCGAGAAAGAAGCGATCAAAGAAGCGTCTTCGGGCGGAGTAGGAGAATGCGGAGGA
GAAGGAGAACGAGCTGATCTAGTATCTCTCCACAATCCAATGCCAACTGACCAACTGGCCATATTCGGAGCAATTTGAAGC
CAATTTCCATCGCCTGGCGATCGCTCCATTCTTGGCTATATGTTTTTCACCGTTACCCGGGGCCATTTTCAAAGACTCGTC
GGCAAGATAAGATTGTGTCACTCGCTGTCTCTCTTCATTTGTCGAAGAATGCTGAGGAATTTCGCGATGACGTCGGCGAGT
ATTTTGAAGAATGAGAATAATTTGTATTTATACGAAAATCAGTTAGTGGAATTTTCTACAAAAACATGTTATCTATAGATA
ATTTTGTTGCAAAATATGTTGACTATGACAAAGAACTGGAATTAAGTGGCATATTTTGCATTGCTTTCCGATAATAGTTGT
ATTTTTATAAAGTAAAGCGTTTGTAAAATAAATACCTTTAATGTATTCTCATTTTCTTATGTATTTATAATGGCAATGATG
ATACTGATGATATTTTAAGATGATGCCAGACCAAAAGGCTTGAATTTCTGCGTCTTTTGCCGAACGCAGTGCATGTGCAAT
TGTTGTTTTTTGGAATATTCAATTTTCGGACTGTCCGCTTTGATTTCAGTTTCTTGGCTTATTCAAAAAGCAAAGTAAAGC
CAAAAAAGCGAGATGGCAATACCAAATGCGGCAAAACGGTAGTGGAAGGAAAGGGGTGCGGGGCAGCGGAAGGAAGGGTGG
GGCGGGGCGTGGCGGGGTCTGTGGCTGGGCGCGACGTCACCGACGTTGGAGCCACTCCTTTGACCATGTGTGCGTGTGTGT
ATTATTCGTGTCTCGCCACTCGCCGGTTGTTTTTTTCTTTTTATGCTGCGCTCTCTCTAGCGCCATCTCGCTTACGCATGC
TCAACGCACCGCATGTTGCCGTTTCCTTTTATGCGTCATTTTGGCTCGAAATAGGCAATTATTTAAACAAAGATTAGTCAA
CGAAAACGCTAAAATAAATAAGTCTACAATATGGTTACTTATTGCCATGTGTGTGCAGCCAACGATAGCAACAAAAGCAAC
AACACAGGTGGCTTTCCCTCTTTCACTTTTTGTTTGCAAGCCGCGTGCGAGCAAGACGGCACGACCGGCAAACGCAATTAC
GCTGACAAAGAGCAGACGAAGTTTTGGCGAAAAACATCAAGGCGCCTGATACGAATGCATTTGCAATAACAATTGCGATAT
TTAATATTGTTTATGAAGCTGTTTGACTTCAAAACACACAAAAAAAAAAAATAAAACAAATTATTTGAAAGAGAATTAGGAA
TCGGACGCTTATCGTTAGGGTAACAACAAGAAATGCTTACTGAGTCACAGCCTCTGGAAAACTGCCGCAAGCCAGAGAGAG
AGAGAAAAAGAGGGAGAGCAGCTTAGACCGCATGTGCTTGTGTGTGAGGCGTCTCTCTCTTCGTCTCTGTTGCGCAAACGC

ATAGACTGCACTGAAAAAATCGATTACCTATTTTTTATGAATGAATATTTGCACTATTACTATTCAAAACTATTAAGATAG
CAATCACATTCAATAGCCAAATACTATACCACCTGAGCGATGCAACGAAATGATCAATTTGAGCAAAAATGCTGCATATTT
AGGACGGCATCATTATAGAAATGCTTCTTGCTGTGTACTTTTCTCTCGTCTGGCAGCTGTTTCGCCGTTATTGTTAAAACC
GGCTTAAGTTAGGTGTGTTTTCTACGACTAGTGAATGCCCTACTAGAAGATGTGTGTTGCACAAAATGTCCCTGGAATAAC
CAATTTGAAGTGCAGATAGCAGTAAACGTAAGCTAATATGAATATTATTTAACTGTAATGTTTTAATATCGCTGGACATTA
CTAATAAACCCACTATAAACACATGTACATATGTATGTTTTGGCATACAATGAGTAGTTGGGGAAAAAATGTGTAAAAGCA
CCGTGACCATCACAGCATAAAGATAACCAGCTGAAGTATCGAATATGAGTAACCCCCAAATTGAATCACATGCCGCAACTG
ATAGGACCCATGGAAGTACACTCTTCATGGCGATATACAAGACACACACAAGCACGAACACCCAGTTGCGGAGGAAATTCT
CCGTAAATGAAAACCCAATCGGCGAACAATTCATACCCATATATGGTAAAAGTTTTGAACGCGACTTGAGAGCGGAGAGCA
TTGCGGCTGATAAGGTTTTAGCGCTAAGCGGGCTTTATAAAACGGGCTGCGGGACCAGTTTTCATATCACTACCGTTTGAG
TTCTTGTGCTGTGTGGATACTCCTCCCGACACAAAGCCGCTCCATCAGCCAGCAGTCGTCTAATCCAGAGAC

The following plasmid was generated by inserting indicated sequence (Ago2 CDS) into the KpnI and SacI sites of **Hy_pAct5C Flag MCS**.

Hy_pAct5C Flag_Ago2_WT

ATGGGAAAAAAAGATAAGAACAAGAAAGGAGGACAGGATAGCGCTGCAGCACCACAGCCCCAGCAGCAGCAAAAGCAACAA
CAACAACGGCAGCAACAACCACAGCAGCTGCAACAACCACAGCAGCTGCAACAACCACAGCAGCTGCAACAACCACAGCAG
CAGCAACAACAACAACCGCATCAGCAACAACAACAAAGTTCGAGACAACAGCCATCCACAAGCTCAGGCGGATCCCGTGCA
TCTGGGTTCCAGCAAGGAGGCCAACAGCAAAAATCCCAAGACGCAGAAGGATGGACTGCACAGAAAAAACAAGGCAAACAG
CAGGTACAAGGGTGGACTAAACAAGGTCAACAAGGTGGCCATCAGCAAGGACGACAAGGGCAAGACGGTGGCTACCAACAG
CGTCCGCCTGGACAACAACAAGGTGGCCATCAGCAAGGACGACAAGGGCAAGAAGGTGGCTACCAACAGCGTCCGCCTGGA
CAACAACAAGGTGGCCATCAGCAAGGACGACAAGGGCAAGAAGGTGGCTACCAACAGCGTCCGTCTGGACAACAACAAGGT
GGCCATCAGCAAGGACGACAAGGGCAAGAAGGTGGCTACCAACAGCGTCCGCCTGGACAACAACAAGGTGGCCATCAGCAA
GGACGACAAGGGCAAGAAGGTGGCTACCAACAGCGTCCGTCTGGACAACAACAAGGTGGCCATCAGCAAGGACGACAAGGG
CAAGAAGGTGGCTACCAACAGCGTCCGCCTGGACAACAACAAGGTGGCCATCAGCAAGGACGACAAGGGCAAGAAGGTGGC
TACCAACAGCGTCCGCCTGGACAACAACAAGGTGGCCATGAGCAAGGACGACAAGGGCAAGAAGGTGGCTACCAACAGCGT
CCGTCTGGACAACAACAAGGTGGCCATCAGCAAGGACGACAAGGGCAAGAAGGTGGCTACCAACAGCGTCCGTCTGGACAA
CAACAAGGTGGCCATCAGCAAGGACGACAAGGGCAAGAAGGTGGCTACCAACAGCGTCCGTCTGGACAACAACAAGGTGGC
CATCAGCAAGGACGACAAGGGCAAGAAGGTGGCTACCAACAGCGTCCGCCTGGACAACAACCAAACCAAACCCAGAGCCAA
GGCCAATACCAATCTCGTGGACCACCTCAGCAACAGCAGGCTGCCCCATTACCATTACCGCCTCAGCCAGCCGGGAGCATT
AAGCGCGGAACAATCGGCAAACCCGGGCAAGTAGGCATCAACTATCTGGACCTTGACCTGTCCAAAATGCCTTCTGTGGCA
TACCACTATGACGTGAAGATCATGCCAGAGCGTCCCAAAAAGTTTTACAGACAGGCATTTGAACAATTTCGGGTGGACCAA
TTGGGCGGAGCAGTTCTTGCCTATGATGGCAAAGCCTCTTGCTACTCGGTGGATAAGCTGCCGTTAAATAGCCAAAATCCG
GAAGTGACTGTGACAGATCGTAATGGTCGTACTTTGCGCTACACGATCGAAATCAAGGAGACTGGTGACTCGACCATTGAC
CTTAAATCGTTAACTACTTATATGAATGACCGAATTTTCGACAAGCCCATGCGAGCAATGCAGTGCGTGGAGGTTGTTTTG
GCTTCACCCTGCCACAACAAAGCCATCCGTGTCGGTCGTTCCTTCTTTAAGATGTCCGATCCTAATAATCGTCACGAACTG
GATGATGGATACGAGGCCCTGGTCGGTCTGTATCAGGCATTTATGCTGGGCGATAGGCCATTTTTAAATGTAGATATATCG
CACAAATCCTTTCCGATTTCAATGCCGATGATCGAATATTTGGAGCGATTTAGCTTAAAGGCTAAAATCAACAATACAACG
AATTTAGATTATTCGCGACGCTTCCTTGAGCCATTCCTCAGAGGCATTAATGTGGTTTACACGCCTCCTCAGTCGTTTCAA
AGCGCTCCAAGGGTATACCGTGTAAATGGTCTTTCTCGTGCCCCGGCCAGCAGTGAGACCTTCGAACACGATGGGAAGAAG
GTCACCATTGCGTCCTACTTCCACAGTCGCAACTACCCATTGAAGTTTCCCCAACTCCATTGTCTGAACGTTGGATCTTCA
ATCAAGAGTATTCTGCTGCCCATCGAGCTATGCAGCATCGAGGAAGGTCAGGCCCTAAACCGCAAGGATGGAGCAACTCAG

GTGGCCAATATGATAAAGTACGCAGCCACATCGACGAACGTGCGAAAGCGCAAGATTATGAACTTGCTGCAATACTTCCAG
CACAACCTGGATCCGACCATCAGTCGCTTTGGCATCCGCATTGCCAACGATTTTATTGTGGTAAGCACCCGCGTCCTAAGC
CCACCTCAGGTTGAATATCATAGTAAGAGGTTTACTATGGTGAAGAACGGGTCGTGGCGCATGGATGGCATGAAGTTTCTG
GAGCCCAAGCCCAAGGCGCACAAGTGTGCGGTCTTGTATTGCGATCCGAGGAGTGGTCGCAAAATGAACTATACCCAGCTG
AATGACTTCGGGAACCTAATAATATCCCAAGGCAAGGCAGTCAACATAAGCTTGGATTCTGATGTGACATACAGACCGTTC
ACGGATGACGAACGCAGCCTAGACACTATTTTCGCGGATCTGAAGCGCAGCCAGCACGATCTGGCAATTGTGATTATTCCT
CAGTTTAGAATTTCTTACGATACAATTAAGCAGAAGGCCGAGCTGCAGCATGGAATTTTGACGCAATGCATTAAGCAGTTC
ACCGTGGAACGAAAGTGTAATAATCAGACGATTGGAAATATTCTACTTAAGATCAACTCCAAGCTGAACGGCATCAACCAC
AAGATCAAGGATGATCCTCGTCTGCCGATGATGAAGAACACCATGTACATTGGAGCCGATGTGACCCATCCCTCTCCCGAT
CAGCGCGAGATTCCCAGTGTGGTCGGAGTAGCAGCCTCACACGATCCCTACGGAGCCAGTTATAACATGCAATATCGTTTG
CAGCGAGGGGCTCTGGAGGAGATTGAGGACATGTTCTCGATTACTTTGGAGCACTTGCGCGTGTATAAAGAGTACCGTAAC
GCCTATCCTGATCATATCATCTACTACCGAGATGGCGTGAGCGACGGCCAGTTTCCGAAAATCAAAAACGAGGAACTGAGG
TGTATTAAACAAGCCTGTGACAAGGTGGGCTGTAAACCCAAGATTTGCTGCGTGATTGTGGTGAAGCGTCATCACACTCGC
TTCTTTCCCAGCGGCGACGTAACGACATCGAACAAGTTCAACAACGTGGACCCCGGAACCGTGGTCGATCGCACCATTGTG
CATCCTAACGAGATGCAGTTCTTCATGGTCAGCCACCAGGCCATCCAGGGCACGGCCAAGCCAACACGATACAATGTGATT
GAGAACACAGGCAATCTTGACATCGACTTGTTGCAGCAGTTGACCTACAACCTGTGCCACATGTTCCCTCGTTGCAATCGC
TCGGTTTCTTATCCGGCTCCGGCCTATTTAGCCCATTTGGTAGCTGCTCGTGGACGCGTTTATCTGACTGGCACCAACAGG
TTCCTGGATTTGAAGAAGGAGTACGCAAAGCGAACGATTGTCCCCGAATTCATGAAGAAAAACCCCATGTACTTTGTCTGA

The following plasmid was generated by introducing the Ago2 V966M mutation (GTG to ATG) to **Hy_pAct5C Flag_Ago2_WT** using site-directed mutagenesis.

Hy_pAct5C Flag_Ago2_MUT
